# Exosomes induce neurogenesis of pluripotent P19 cells

**DOI:** 10.1007/s12015-023-10512-6

**Published:** 2023-02-22

**Authors:** Antje Anji, Briana Anderson, Feroz Akhtar, David A. Meekins, Takashi Ito, Srinivas Mummidi, Meena Kumari

**Affiliations:** 1 Department of Anatomy and Physiology, College of Veterinary Medicine, Kansas State University, Manhattan, KS 66506 USA; 2 Present Address: Kansas Department of Health and Environment, Bureau of Epidemiology and Public Health Informatics, Topeka, KS 66612 USA; 3 Present Address: School of Physical Therapy, Arkansas Colleges of Health Education, Fort Smith, AR 72916 USA; 4 Present Address: Department of Life Sciences, Texas A&M University, San Antonio, TX 78224 USA; 5 Department of Diagnostic Medicine/Pathobiology, College of Veterinary Medicine, Kansas State University, Manhattan, KS 66506 USA; 6 Present Address: Department of Biochemistry and Molecular Biophysics, Kansas State University, Manhattan, KS 66506 USA; 7 Department of Chemistry, College of Arts and Sciences, Kansas State University, Manhattan, KS 66506 USA

**Keywords:** Pluripotent P19 cells, Exosomes, Neurogenesis, Small RNA-Seq, Non-coding RNAs

## Abstract

Exosomes play a role in tissue/organ development and differentiation. Retinoic acid induces differentiation of P19 cells (UD-P19) to P19 neurons (P19N) that behave like cortical neurons and express characteristic neuronal genes such as NMDA receptor subunits. Here we report P19N exosome-mediated differentiation of UD-P19 to P19N. Both UD-P19 and P19N released exosomes with characteristic exosome morphology, size, and common protein markers. P19N internalized significantly higher number of Dil-P19N exosomes as compared to UD-P19 with accumulation in the perinuclear region. Continuous exposure of UD-P19 to P19N exosomes for six days induced formation of small-sized embryoid bodies that differentiated into MAP2-/GluN2B-positive neurons recapitulating RA-induction of neurogenesis. Incubation with UD-P19 exosomes for six days did not affect UD-P19. Small RNA-seq identified enrichment of P19N exosomes with pro-neurogenic non-coding RNAs (ncRNAs) such as miR-9, let-7, MALAT1 and depleted with ncRNAs involved in maintenance of stem cell characteristics. UD-P19 exosomes were rich with ncRNAs required for maintenance of stemness. P19N exosomes provide an alternative method to genetic modifications for cellular differentiation of neurons. Our novel findings on exosomes-mediated differentiation of UD-P19 to P19 neurons provide tools to study pathways directing neuron development/differentiation and develop novel therapeutic strategies in neuroscience.

**Graphical Abstract:**

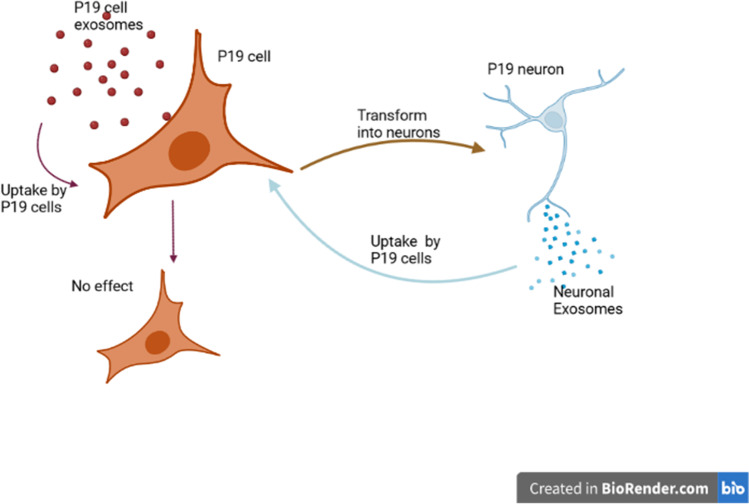

**Supplementary Information:**

The online version contains supplementary material available at 10.1007/s12015-023-10512-6.

## Introduction

Intercellular communication through exosomes facilitates cell differentiation from progenitor/stem cells [1–3]. Exosomes are small microvesicles with a diameter ranging from ~ 30 to 150 nm originating in endosomes of cells and are released by all cells in vivo and in vitro [4, 5]. Exosomes exert their effects on recipient cells through their unique bioactive cargo of proteins, lipids, small RNAs, and/or metabolites that often reveal their cell origin (donor/parent cell) [6–8]. The composition of exosomal cargo changes with physiology and pathology of the parent cells [9–11]. In addition, exosomes contain certain proteins and lipids that reflect their endosomal origin and are hence used as markers for their characterization. Neurons are terminally differentiated and are highly specialized cells. Two studies demonstrated neurogenic effects of neuronal exosomes. The hiPSC-derived neuronal exosomes induce cell proliferation and neurogenesis through activation of signaling cascades [12]. In a 2^nd^ study, cyclin D1 enriched N2A neuronal exosomes induced neuron differentiation of mouse embryonic stem cells [13]. Both studies utilized medium containing B27 that contains retinol, a precursor of retinoic acid (RA). RA is an inducer of neurogenesis [14] and thus may have primed the cells for neuronal differentiation. In the present study, we used P19 cells to investigate the role of neuronal exosomes in differentiation of pluripotent P19 cells into neurons in the absence of B27 media supplement.

P19 cells, a pluripotent embryonal cell line derived from mouse teratocarcinoma, can differentiate into cells of all three germ layers upon stimulation with various agents [15]. For instance, exposure to retinoic acid for four days induces differentiation of P19 cells to P19 neurons, a process reflective of embryonic neurogenesis in vitro [16]. P19 cells thus offer an ideal model system to examine role of intercellular communication through exosomes in neuronal differentiation. In this manuscript, we characterized exosomes purified from the conditioned medium of undifferentiated P19 cells (UD-P19) and P19 neurons (P19N) by differential ultracentrifugation. Purified P19N exosomes were internalized by UD-P19 and P19N. Continuous exposure to P19N exosomes induced differentiation of UD-P19 to MAP2- and GluN2B-positive neurons with long neurites. The number of neurons increased with increase in exosome exposure time. Under similar culture conditions, exposure to UD-P19 exosomes did not induce UD-P19 differentiation into neurons. Since exosomal RNAs modulate gene expression during differentiation [17], we analyzed UD-P19 and P19N exosome RNAs by small RNA-Seq. P19N exosomes were enriched with pro-neurogenic ncRNAs and depleted with pro-stemness/cell proliferation ncRNAs. In contrast, UD-P19 exosomes were particularly rich in pro-stemness and stem cell proliferation ncRNAs. These data demonstrate the importance of intercellular communication via exosomes and their rich cargo of pro-neurogenic ncRNAs in differentiation of neurons. Neuronal differentiation mediated by exosomes in a culture dish ‘on demand’ provides a tool to study embryonic neurogenesis pathways, identify key molecules, and provides new opportunities for therapeutic interventions.

## Materials and Methods

### Materials

Alpha-MEM (catalog # M4526), 100 × antibiotic–antimycotic solution, retinoic acid, 5‐fluorouridine, uridine, and all general chemicals were purchased from MilliporeSigma, MO; neurobasal medium, N2 and B27 supplements, Dulbecco’s phosphate buffered saline (DPBS) (catalog #14,190–144) from Invitrogen; copper grids, nickel grids, mica wafers, metal specimen discs, formaldehyde, glutaraldehyde, and uranyl acetate from Electron Microscopy Sciences, PA; gold conjugated secondary antibody from Jackson ImmunoResearch Inc., PA; 40% acrylamide/Bis solution (29:1), bovine serum standard, Bradford protein assay dye reagent concentrate, nitrocellulose membrane from BioRad, CA; Dil (1,1'-Dioctadecyl-3,3,3',3'-Tetramethylindocarbocyanine Perchlorate ('DiI'; DiIC18(3)) (#D282), The Pierce 660-nm protein assay kit, Whatman filter paper #1, Pierce™ ECL Plus substrate from ThermoFisher, MA. The miRCURY™ RNA isolation kit was purchased from Exiqon, Inc. (now Qiagen) and fetal bovine serum from Atlanta Biologicals (now part of R&D Systems, Inc., MN; and ECL Plus reagent from Lumigen, MI. Antibodies used in this study are shown in Table 1.

**Table 1:** List of primary antibodies used in the study

Antibody	Immunogen	Manufacturer	Concentration	RRID	Host
CD9	Raised against amino acids 101–210 of CD9 of human origin	Santa Cruz Biotechnology, TX Cat # sc-13118	1:1,000	AB_627213	Mouse monoclonal
CD63	Raised against full length CD63 of human origin	Santa Cruz Biotechnology, TX Cat # sc-5275	1:1,000	AB_627877	Mouse monoclonal
CD81	Raised against raised against OCI-LY8 cells	Santa Cruz Biotechnology, TX Cat # sc-23962	1:1,000	AB_627192	Mouse monoclonal
tumor susceptibility gene 101 (tsg101)	Raised against amino acids 1–138 representing full length tsg 101 of mouse origin	Santa Cruz Biotechnology, TX Cat # sc-7964	1:1,000	AB_671392	Mouse monoclonal
Flotillin-1	Clone 18 Raised against amino acids 312–428 Flotillin-1 of mouse origin	BD Transduction Laboratories™, San Jose, CA Cat # sc-365214	1:500	AB_398139	Mouse monoclonal
Cytochrome C	Raised against amino acids 1–104 of cytochrome c of equine origin	Santa Cruz Biotechnology, TX Cat # sc-13156	1:1,000	AB_627385	Mouse monoclonal
Dicer1	Raised against synthetic peptide surrounding amino acid 1902 of human dicer	BioVision Milpitas, CA Cat # 3697–100	1:500	AB_2093067	Rabbit polyclonal
Argonaute-2 (C34C6)	Raised against a synthetic peptide corresponding to mouse Argonaute 2	Cell Signaling Technology, Danvers, MA Cat # #2897 T	1:1,000	AB_2096291	Rabbit monoclonal
MAP2	Raised against purified microtubule-associated protein from rat brain	Chemicon (now part of Millipore Sigma) Cat # AB-5622	1:500	AB_91939	Rabbit polyclonal
GluN2B (also known as NMDAR2B)	Raised against C-terminal fusion protein of NMDAR2B (30 kDa)	Millipore (now Millipore Sigma) Cat # AB1557P	1:500	AB_90772	Rabbit polyclonal

### P19 Cell Culture

Undifferentiated P19 (UD-P19) cells (RRID: CVCL_2153) were cultured in α-MEM supplemented with 10% fetal bovine serum (FBS) in 75 cm^2^ flasks using our protocol described previously [18]. UD-P19 culture conditions were optimized for exosome isolation in the absence of FBS due to presence of exosomes in the serum. Based upon initial experiments, UD-P19 were passaged at 1:5 ratio (~ 50% confluency) that resulted in ~ 70% confluency 24 h after plating in α-MEM/10% FBS. Next day, cells were washed with pre-warmed 1 × Dulbecco phosphate buffered saline (DPBS) and fed α-MEM containing 1x N2 supplement. After 24 h, conditioned medium (10 mL per flask × 6 = 60 mL) was collected to isolate exosomes (Supplementary Figure S1).

### Differentiation of P19 Neurons and Culture

UD-P19 were induced to differentiate into P19 neurons (P19N) by culturing them in α-MEM/5% FBS/retinoic acid (RA) using our previously described protocol [18]. Briefly, free floating embryoid bodies obtained after 4 days of RA treatment of UD-P19 were plated on poly-L-lysine coated 75 cm^2^ flasks in neurobasal medium containing 0.5 mM glutamine, B27 and N2 supplements for 8 to 16 days in vitro (Supplementary Figure S1). Embryoid bodies formed were of varied sizes and hence it was difficult to predict the number of cells in embryoid bodies plated in each flask. However, based upon the surface area of a 75 cm^2^ flask covered by neurons and their neurites, we referred to P19 neuronal cultures as ~ 70% confluent. After plating, P19N continue to proliferate. Cell proliferation was inhibited by exposure to 5‐fluorouridine and uridine for 24 h on days 3 and 8 (after collecting conditioned medium on day 8) of culture and cells were fed fresh medium 24 h later. Conditioned medium (10 mL per flask × 3 = 30 mL) was collected on day 8, and on every alternative day thereafter to isolate exosomes (Supplementary Figure S1).

### Isolation of Exosomes

Conditioned medium (CM) collected from cultured cells was processed immediately for exosome isolation using differential centrifugation method described by Street and colleagues with one exception [19] and personal communication with authors. Beckman SW 55Ti rotor [r_min_ = 60.8; r_max_ = 108.5; r_av_ = 84.6 mm; rotor capacity = 5 × 6 mL] was used in lieu of 45Ti rotor for all high-speed centrifugation steps in XL-80 K Beckman ultracentrifuge. The speed and time of run was therefore configured accordingly for SW 55Ti rotor (Supplementary Figure S2). CM was initially centrifuged at 2,500 g, 10 min at 4ºC in a tabletop low speed centrifuge (Allegra, Beckman Coulter, Inc.). Pellet containing cells was discarded. Supernatants were recovered and centrifuged in SW 55Ti at 20,000 g, 10 min at 4ºC [14,500 rpm; k-factor = 697.8; RCF_avg_ = 19,933; RCF_max_ = 25,550]. Pellets were discarded and supernatants were centrifuged at 200,000 g, 33 min at 4ºC [45,900 rpm; k-factor = 69.6; RCF_avg_ = 199,742; RCF_max_ = 256,020]. Supernatants were discarded and pellets containing exosomes were recovered from all six tubes using ice-cold DPBS by gentle pipetting. Exosomes from all six tubes were pooled and centrifuged again at 200,000 g, 33 min at 4ºC (wash step). Supernatant was discarded and pellet containing washed exosomes was suspended in 100-150µL DPBS by gentle pipetting. Protein concentration of exosomes was determined using the Pierce 660-nm protein assay according to instructions of the supplier. Samples were either processed immediately for an experiment or stored at -80 ºC until further use.

### Characterization of Exosomes

The desiccated exosomes were characterized by transmission electron microscopy and atomic force microscopy; and the hydrated exosomes by nanoparticle tracking analysis (NTA).

#### Transmission Electron Microscopy (TEM)

Freshly purified exosomes were diluted with DPBS and 4 µL volume was deposited on formvar coated 200 mesh copper grids. After 2 min, grids were blotted dry on Whatman filter paper #1. The grids were inverted over a drop of 2% aqueous uranyl acetate. After 2 min, grids were blotted dry on Whatman filter paper #1, and air dried. All grids were examined under transmission electron microscope, FEI Tecnai G2 Spirit BioTWIN (FEI Company, Hillsboro, Oregon, USA) at an acceleration voltage of 80 kV and digital images were captured with scale bar using a GATAN digital imaging system (Gatan, Inc., CA). Exosome size was measured using GATAN microscopy suite (Gatan, Inc., CA). The diameter of 120 exosomes per sample was manually marked and calculated using “line annotation with length” standard tool of GATAN software. The diameter of UD-P19 and P19N exosomes was analyzed using unpaired two-tailed t-test (GraphPad Prism, version 9.2.0).

#### Atomic Force Microscopy (AFM)

A sheet of muscovite mica (V1, 9.5 mm in diameter) mounted on a metal specimen disc was used as a substrate. Freshly purified exosomes were diluted in DPBS (5 ng/10 µL), and 10 µL of the diluted solution was deposited onto a freshly-cleaved mica sheet. The resulting samples were air-dried. Salt (in DPBS) crystallized on mica was removed by careful washing the dried exosomes once with milliQ water. Exosomes were air-dried and examined using a Digital Instruments Multimode Atomic Force Microscope with Nanoscope IIIa electronics (Digital Instruments, Santa Barbara, CA). Tapping-mode AFM images (512 pixel × 512 pixel) were obtained in air using AFM probes purchased from Asylum (Model HQ-150-Au). These images (1 × 1 µm^2^) were analyzed using the particle analyzer of the ImageJ software [20] to assess particles larger than 78 pixel^2^, which corresponds to 19.5 nm in diameter for circular particles. Similar AFM images were obtained for four different biological preparations of exosomes purified on different days. The total number of particles analyzed for UD-P19 and P19N exosome preparations were 890 and 1,330 respectively.

#### Nanoparticle Tracking Analysis (NTA)

NTA was performed using NanoSight LM10 equipped with a 405-nm laser, a Hamamatsu digital camera, and a sample chamber lined with a Viton fluoroelastomer O-ring (NanoSight, Amesbury, United Kingdom). The camera level and detection threshold were set at values of 13 and 5 respectively. All measurements were performed at 37 °C. Monodisperse polystyrene beads (size 200 nm) suspended in DPBS were used to calibrate the instrument. Exosomes suspended in DPBS (1 µg/mL) were injected into the sample chamber with sterile 1 mL syringe (BD Bioscience, New Jersey, USA) until the liquid reached the tip of the nozzle. Once temperature was at 37 °C, Brownian motion of exosomes was captured by five repeated 60 s video recordings with shutter speed of 31.48, frame rate of 30 frames/second, and gain adjustments to 366 using NTA 3.3 Dev Build 3.3.301 software (Malvern Panalytical Ltd., Worcestershire, United Kingdom). The particle size distribution profiles and concentration measurements of the exosomes in solution were calculated automatically by NTA 3.3 software. The average mode size (diameter in nm) was calculated using data from five technical repeats per sample. The average mode size from five different biological preparations of exosomes purified on different days was analyzed using unpaired two-tailed t-test (GraphPad Prism, version 9.2.0). The exosome concentration was calculated using data from 12–14 different biological preparations of exosomes purified on different days and expressed as exosome numbers per volume of conditioned medium (exosome number/mL CM). Statistical analysis was performed by unpaired two-tailed t-test.

### Authentication of Exosomes

#### Immunoelectron Microscopy

Exosomes were deposited on the carbon coated 200 mesh nickel grids (EMS) as above. After 2 min, excess fluid was removed and exosomes were fixed in Trump’s fixative for 10 min [21]. After washing with milliQ water (three times; 3 min each wash), grids were incubated with 50 mM acetate solution for 10 min to neutralize aldehyde groups. After washing with DPBS (three times; 3 min each wash), exosomes on grids were blocked in 5% donkey serum in DPBS for 30 min. Grids were incubated with anti-CD63 (1:250 dilution; 1 h) or DPBS in lieu of primary antibody (negative control) in a humid atmosphere. After washing with DPBS-Tween (DPBS containing 0.05% Tween 20) (six times; 3 min each wash), grids were first incubated for 30 min with AffiniPure Fab fragment and then with 18 nM gold conjugated AffiniPure donkey anti-mouse secondary antibody (1:20 dilution; 30 min). Grids were washed in DPBS-Tween as above (eight times; 3 min each wash) and negatively stained with 2% aqueous uranyl acetate. Grids were air dried and examined under transmission electron microscope (FEI Tecnai G2 Spirit BioTWIN) at an acceleration voltage of 80 kV. Digital images were captured with scale bar using a GATAN digital imaging system. All steps involving washings and incubations were carried out by inverting grids over a drop of water, buffer or diluted antibody solutions and performed at room temperature.

#### Western Blotting

Western blot conditions in terms of lysis buffer for solubilizing exosomes, amount of exosomal proteins required for each antibody, and dilution of antibodies were optimized using fetal cortical neuronal (FCN) lysates and exosomes to detect the respective cognate protein (Table 1, Supplementary Table TS2, Supplementary Figure S3). Optimized Western blot conditions were used to detect specific marker proteins in UD-P19 and P19N exosomes. Cell lysates (nuclei-free) from UD-P19, P19N, and primary mouse cortical neurons were included as controls. Protein concentration in lysates was determined using Bradford method [22].

Exosomes (30 to 100 µg protein) were centrifuged at 200,000 g, 33 min at 4 ºC (as above) in conical Beckman centrifuge tubes. Exosome pellets were solubilized in appropriate lysis buffer, mixed with 5X Laemmli’s gel loading dye, and heat-denatured in boiling water for 3 min prior to storage until electrophoresis (Supplementary Table TS2, Supplementary Figure S3). Proteins were separated on appropriate pore-size SDS-PAGE depending upon the molecular mass of the target protein of interest (Supplementary Table TS2). Separated proteins were blotted onto nitrocellulose membrane that was stained with Ponceau S stain with the exception of CD63 membrane. After capturing images of Ponceau stained membrane with Kodak EDAS imaging system, it was de-stained and blocked for 2 h in TBST (5% milk in 20 mM Tris–HCl buffer pH 7.5 containing 150 mM NaCl, 0.1% Tween 20). Incubation with primary antibody (Table 1) in 5% milk in TBST was overnight at 4 °C prior to incubation with HRP-conjugated Affinipure goat anti-mouse secondary antibody (1:2,500 dilution in 5% milk in TBST; Jackson ImmunoResearch) or HRP-conjugated Affinipure goat anti-rabbit secondary antibody (1:2,500 dilution; Jackson ImmunoResearch) for 2 h at room temperature. Washed membrane was exposed to ECL plus solution for 5 min for detection of immunoreactive bands on PhosphorImager. Data was analyzed using ImageQuant software. Two exceptions made to detect CD63 in UD-P19 and P19N lysates by Western blotting were: 1) nitrocellulose membranes were incubated with Pierce™ ECL Plus Western blotting substrate (ThermoScientific) for 5 min; and 2) membranes were scanned using Azure 300 gel imaging scanner for detection of immunoreactive bands.

### Fluorescence Labeling and Cellular Uptake of P19N Exosomes

Exosomes were labeled with a lipophilic photostable red fluorescent membrane dye, Dil. Briefly, a 1 mM solution of Dil was prepared in absolute ethanol and stored at room temperature in dark. Exosomes suspended in 1 mL DPBS were incubated with 1 µL of 1 mM Dil solution for 18 min at room temperature in dark. At the end of the incubation, Dil incubated exosomes were mixed with 4 mL of DPBS and centrifuged at 200,000 g, 33 min at 4 ºC in SW 55Ti rotor in Beckman ultracentrifuge. Exosome pellet (dark pink color) was suspended in DPBS and ultracentrifuged as in the previous step. Washed exosome pellet was suspended in 100 µL of DPBS and an aliquot was examined by NanoSight. Following estimation of exosome protein concentration, exosomes were mixed with NB/N2 medium to obtain 40 µg/mL concentrations and filtered through 0.2µ syringe filter. As a control, 1 mL DPBS was incubated with 1 µL of 1 mM Dil and processed simultaneously the same way as Dil-labeled exosomes. For simplicity, we express exosomes used for cellular uptake and biological effects in exosome microgram protein. Based upon calculations, 20 µg P19N exosome protein contained ~ 6.82e + 09 exosomes equivalent to ~ 9.44 mL CM volume. Forty micrograms (40 µg) exosome protein contained ~ 13.64e + 09 exosomes equivalent to ~ 18.88 mL CM volume. Following suspension of 20 µg exosome protein per mL culture medium (or 40 µg/mL), 350 µL was fed to cells per well. This volume (350 µL) of exosomes was equal to 7 µg exosome protein = 2.387e + 09 = 3.304 mL CM volume for 20 µg/mL exosome suspension and 14 µg exosome protein = 4.774e + 09 = 6.608 mL CM volume for 40 µg/mL exosome suspension.

Exosome uptake study was performed using P19N and UD-P19. P19N were cultured in four wells in 8-well poly-L-lysine coated plates for 8 days (Supplementary Figure S1). UD-P19 were seeded at a density of 4,000 cells per well in four wells of 8-well plates two days prior to incubation with exosomes. Both P19N and UD-P19 were cultured in such a manner so that they were ready for incubation with exosomes simultaneously. Prior to addition of exosomes to the medium, 8-day old P19N and UD-P19 were washed with culture medium. Treated cells were incubated with 350 µL of Dil-labeled exosomes (20 or 40 µg/mL) for 0 h, 6 h, 12 h, and 24 h. Cells in control wells received 350 µL of Dil/DPBS processed in the same way as exosomes and incubated for 24 h time duration only. Incubation with exosomes was staggered so that cells in all wells could be processed simultaneously. At the end of the incubation with exosomes, cells were washed twice with DPBS and fixed in freshly prepared 4% formaldehyde for 10 min at room temperature. After washing three times with autoclaved milliQ water and once with DPBS containing DAPI (10 min/wash), cells were suspended in DPBS and examined under confocal microscope (Carl Zeiss, LSM-700). Internalization of Dil-labeled exosomes was evaluated by capturing images with 20X (EC Plan-Neofluar 20X/0.5 M27) or 40X oil (EC Plan-Neofluar 40x/1.30 Oil M27) objective. Intracellular localization of Dil-exosomes was determined by z-stacking (optical slicing) of cells and these images (z-stacks) were processed into maximum intensity projections in Zeiss Zen Black. Time-dependent internalization of Dil-labeled exosomes was quantitated by calculating the corrected total cell fluorescence (CTCF) using ImageJ. The following formula was used to calculate the CTCF: CTCF = integrated density – (area of selected cell x mean fluorescence of background readings). Statistical analysis of CTCF among different time points for each cell type was performed by ordinary one-way ANOVA and Tukey’s multiple comparisons test (GraphPad Prism, version 9.2.0).

### Biological Effects of P19N Exosomes Upon Internalization by UD-P19

To examine effects of P19N exosomes internalized by UD-P19, exosomes were purified from CM of 8d old P19N, and washed once with DPBS (see exosome purification method above). Washed exosomes were suspended in α-MEM/N2 medium to obtain 40 µg exosomes per mL medium concentration. Suspended exosomes were filtered through 0.2µ syringe filter and incubated with UD-P19 for up to a period of six days.

On day 1, UD-P19 (4,000 cells in 350 µL α-MEM/10% FBS per well) were seeded in four wells per 8-well plate (six 8-well plates/experiment) and cultured for 2 days. On day 3, FBS containing medium was changed to α-MEM containing 1 × N2 supplement and 1 × antibiotic/anti-mycotic solution. Cells were washed twice with α-MEM/N2 medium and fed medium containing P19N exosomes every 48 h (350 µL of exosomes in α-MEM/N2 medium per well). Control cultures received fresh α-MEM/N2 medium (350 µL/well) every 48 h. Cultures were terminated after 2, 4, and 6 days of exposure to P19N exosomes. Cells were rinsed in DPBS and fixed in freshly prepared 4% formaldehyde/DPBS for 10 min at room temperature. Initially, phase contrast images of cells were captured using LWD 20x/0.4NA Ph1 ADL objective of Nikon Eclipse TS100 microscope equipped with cannon camera. Subsequently cells were stained for neurite marker, MAP2 protein by immunocytochemistry [23]. Please refer to previous method (Fluorescence labeling and cellular uptake of P19N exosomes) for information on number of exosomes in 350 µL volume.

For MAP2 staining, cells fixed in formaldehyde were washed with water (2 × 10 min/wash) and permeabilized with 0.1% Triton X100 in DPBS for 5 min. After three washes with DPBS (10 min/wash), cells were blocked in 0.22 micron filtered 5% donkey serum in DPBS for 2 h at room temperature and then overnight with anti-MAP2 (1:500 dil in 1% donkey serum in DPBS) at 4 °C in a humid atmosphere. Next day, cells were washed three times with DPBS (10 min/wash) and incubated with Alexa 488 conjugated donkey anti-rabbit secondary antibody (1:1,000 dil in 1% donkey serum in DPBS) for 2 h at room temperature. After DPBS wash (3 × 10 min/wash; last wash with DAPI containing DPBS), cells were mounted in FluorSave (MilliporeSigma) and examined under confocal microscope (Carl Zeiss, LSM-700). Images were captured as tiles, tiles with z-stacks, or z-stacks using Zen Black software. All tiled images spanned a region of 2,560 × 2,560 microns. The z-stacks of tiled images of cells were captured using 10X (Plan-Neofluar 10x/0.30) objective and stitched using Stitch feature of Zen software. The z-stacks of cells were captured with 40X oil (EC Plan-Neofluar 40x/1.30 Oil M27) objective. All z-stacks were processed into maximum intensity projections in Zeiss Zen Black.

Method for GluN2B staining was similar to MAP2 staining in terms of antibody dilution and their incubation time with the exception that the secondary antibody was Alexa 594 conjugated donkey anti-rabbit. Images were captured with Zeiss confocal microscope using Zen Black software and processed using Zen blue. A region of interest was cropped using ROI function of Zen blue software.

### Biological Effects of UD-P19 Exosomes upon Internalization by UD-P19

As a control, effect of UD-P19 exosomes internalization by UD-P19 was examined using a similar treatment paradigm as for P19N exosomes. First, maximum uptake of UD-P19 exosome by UD-P19 was determined. UD-P19 exosomes labeled with Dil (as above) were incubated with UD-P19 (4,000 cells/well) at two concentrations (20 µg/mL and 40 µg/mL; 350 µL/well) for 24 h. Cells in control wells received 350 µL of Dil/DPBS processed in the same way as exosomes. At the end of the incubation with exosomes, cells were fixed, counterstained with DAPI and examined under confocal microscope (Carl Zeiss, LSM-700). Images of cells with internalized Dil-exosomes were captured as z-stacks with 40X oil (EC Plan-Neofluar 40x/1.30 Oil M27) objective and z-stacks were processed into maximum intensity projections in Zeiss Zen Black. Concentration-dependent internalization of Dil-labeled exosomes was quantitated by calculating CTCF using ImageJ. Statistical analysis of CTCF between two exosome concentrations was performed by unpaired two-tailed t test (GraphPad Prism, version 9.2.0). For simplicity, we express UD-P19 exosomes used for cellular uptake and biological effects in exosome microgram protein. Based upon calculations, 20 µg UD-P19 exosome protein contained 7.82e + 09 exosomes equivalent to ~ 13 mL CM volume. Forty micrograms (40 µg) exosome protein contained ~ 15.64e + 09 exosomes equivalent to ~ 26 mL CM volume. Following suspension of 20 µg UD-P19 exosome protein per mL culture medium (or 40 µg/mL), 350 µL was fed to cells per well. This volume (350 µL) of exosomes was equal to 7 µg exosome protein = 2.737e + 09 = 4.55 mL CM volume for 20 µg/mL exosome suspension and 14 µg exosome protein = 5.474e + 09 = 9.1 mL CM volume for 40 µg/mL exosome suspension.

To determine biological effects of UD-P19 exosomes, UD-P19 (4,000 cells/well) plated in 8-well plates were cultured for 24 h in α-MEM/10% FBS prior to incubation with freshly isolated UD-P19 exosomes (350 µL/well). Two concentrations (20 µg/mL and 40 µg/mL) of UD-P19 exosomes were used for 6 days with feeding fresh exosomes to cells every 48 h. Control cultures received fresh medium every 48 h. At the end of 6d, cells were fixed in 4% formaldehyde/DPBS for 10 min at room temperature. After washing, cells were suspended in DPBS and differential interference contrast (DIC) images of cells were captured using Leica DMI6000 B inverted microscope (equipped with Hamamatsu 1394 ORCA-ERA camera and IP Lab software version 4.0.4) with 40X  dry (HCX PL FLUOTAR 40x/0.75 PH2) objective.

UD-P19 plated at a lower density (800 cells/well) were incubated with UD-P19 exosomes (350 µL of 40 µg/mL UD-P19 exosomes in α-MEM/N2 medium) for 6d with change of medium containing freshly isolated exosomes every 48 h. Cells in control wells were fed 350 µL fresh α-MEM/N2 medium every 48 h. At the end of 6d, cultures were washed and fixed in formaldehyde as above. After washing, cells were permeabilized in 0.1%Triton X100/DPBS for 25 min, washed twice with DPBS, and stained with Alexa Fluor™ 647 Phalloidin (1:20 dil in DPBS) for 30 min at room temperature. Cells were washed, counterstained with DAPI and examined under confocal microscope (Carl Zeiss, LSM-700). Images of stained cells were captured with 40X oil (EC Plan-Neofluar 40x/1.30 Oil M27) objective and processed using Zeiss Zen Blue.

### Isolation of Exosomal RNAs and Initial RNA Analysis

Exosome pellets from UD-P19 and P19N were suspended in 100 µL of DEPC water and processed immediately for isolation of RNA using miRCURY™ RNA isolation kit (Exiqon, Inc.; now Qiagen) according to the instructions of the Supplier. Purified RNA was eluted using 50 µL of DEPC water. RNA concentration was measured using NanoDrop™ 8000 spectrophotometer (ThermoFisher Scientific). Initially, size of exosomal RNAs was determined by separating RNA samples on microfluidics-based 2100 BioAnalyzer system (Agilent Technologies, Santa Clara, CA) using Agilent Small RNA kit (reorder number 5067–1548) according to the manufacturer’s protocol. Small RNA ladder (20, 40, 60, 80, and 150 nt long) was used as a reference. RNA ladder and RNA (1 µL per sample) were heat denatured (70 °C/2 min), quickly cooled on ice prior to loading on microfluidic chip and electrophoresis on BioAnalyzer. RNA marker (4 nt long) was loaded in all wells to manually align the sample, if necessary, with the RNA internal marker in the electropherogram.

### Radiolabeling Exosomal RNAs and Analysis on Denaturing Polyacrylamide Gel

Exosomal RNAs (100 ng per sample) were dephosphorylated by incubation with shrimp alkaline phosphatase (NEB) at 37 °C/60 min. At the end of the incubation, phosphatase was inactivated by addition of EDTA and heating at 65 °C for 20 min. Without any purification, dephosphorylated RNAs were end-labeled with ^32^P-γ-ATP (20 µCi/sample) (specific activity: 3,000 Ci/mMol; PerkinElmer, USA) using T4 polynucleotide kinase (NEB). The same protocol was used to end-label double-stranded DNA molecular weight markers (10 and 50 bp ladders from Gibco-BRL and Promega Inc., USA respectively). The ΦX174 DNA/HinfI dephosphorylated DNA molecular weight markers (Promega) were end-labeled by incubation with 10 µCi of ^32^P-γ-ATP in the presence T4 polynucleotide kinase for 10 min at 37 °C. A known volume of labeled RNA and DNA molecular weight markers were independently dried under vacuum, mixed with formamide based gel loading dye, heat-denatured (90 °C/10 min) and separated on 10% sequencing gel under denaturing conditions. Gels were dried, exposed to PhosphorImager screen and scanned next day on PhosphorImager (GE, Boston, MA, USA).

### Small RNA-Seq Library Construction

RNAs (200 ng) from exosomes of UD-P19 (three biological replicates) and P19N (8, 10, and 12 days old) were processed to generate double stranded barcoded cDNA libraries using NEBNext® Multiplex Small RNA Library Prep Set for Illumina (NEB) according to instructions of the supplier. These six samples were the same samples analyzed by end-labeling experiment above. Briefly, after ligation of 3’ SR adaptor to exosomal RNAs, reverse transcription primer was hybridized. After ligation of 5’ SR adaptor, modified RNAs were reverse transcribed using ProtoScript II reverse transcriptase by incubation at 50 °C for 1 h. Following addition of LongAmp Taq master mix with SR primer for Illumina and index primer (unique for each RNA sample) to RT reaction mix, cDNAs were amplified by PCR [step 1: 94 °C/30 s × 1; step 2: 94 °C/15 s, 62 °C/30 s, 70 °C/15 s × 12; step 3: 70 °C/5 min × 1, step 4: 4 °C/hold]. The cDNA library from each sample had a unique barcode through the use of index primers during PCR amplification. The barcoded cDNA libraries were purified using Monarch PCR and DNA cleanup kit (NEB) and quality control was measured on BioAnalyzer using Agilent DNA High Sensitivity chip. All libraries were quantified with KAPA Library Quantification kit for Illumina (KAPA Biosystems, #KK4824) using the BioRad CFX96 Real Time PCR system. Subsequently, libraries were pooled in equimolar amounts and sequenced at K-State Integrated Genomics Facility with NextSeq mid output 150 cycles kit, PE 75 bp, (Illumina, #FC-404–2001) on Illumina NextSeq 500 platform according to manufacturer's recommendations to provide full to partial coverage of RNAs in the samples.

### Small RNA-seq Data Analysis

The 3’ adaptor sequences were trimmed, and low-quality reads were removed during the FASTQ file generation. Paired sequences for each sample were generated using the CLC Genomics Workbench (Qiagen®) and then concatenated. The default parameters in the Workbench were used to analyze the samples and derive the counts. The ncRNA database (GRCm38.p6; https://www.gencodegenes.org/) was used to annotate RNAs in all samples. Enrichment of ncRNAs was determined by using the empirical analysis of DGE tool of the Workbench which implements the 'Exact Test' for two-group comparison as incorporated in the edgeR Bioconductor package. The test uses the raw counts, and implicitly carries out normalization and transformation of these counts. It is based on the assumption that the count data follows a Negative Binomial distribution. Annotation of the differentially enriched ncRNAs was performed using the Biomart tool of Ensembl database to identify their gene type, gene description, transcript stable ID, transcript name, RefSeq ID, transcript type, chromosomal location and coordinates, and strand information. Additional annotations were generated from the UCSC genome browser. Annotated ncRNAs were manually segregated into different classes of ncRNAs with emphasis on up- or down- regulation. Publicly available resources such as Mouse Genome Informatics (MGI) and PubMed were used to determine the location and function of the differentially enriched ncRNAs important in neural stem cell characteristics and neurogenesis. MicroRNA targets were predicted and prioritized using the tools available in miRDB (www.mirdb.org). The Morpheus software (https://software.broadinstitute.org/morpheus/) was used to generate a heat map with two-way hierarchical clustering of differentially enriched transcripts. The enrichment analysis was performed using differentially expressed ncRNAs to identify GO:TERMS using MGI (MGI 6.18 version) on March 11, 2022 [24–26].

### Validation of RNA-Seq Data

MicroRNA-9 (miR-9) was detected only in P19N exosomes. This observation was validated by real-time PCR method using TaqMan™ Advanced miRNA cDNA Synthesis kit (Applied Biosystems) according to the instructions of the manufacturer. The housekeeping control, has-miR-361-5p, was amplified simultaneously. The RT-qPCR analysis of miR-9 and hsa-miR-361-5p (housekeeping control) were conducted using TaqMan miRNA assay in triplicate, each 20 µL reaction mixture included 5 µL of 10x-diluted reverse transcriptase product. Reactions were run on QuantStudio 12 K Flex Real Time system [step 1: 95 °C/20 s—1 cycle; step 2: 95 °C/1 s and 60 °C/20 s—40 cycles]. To account for possible difference in the amount of starting RNA and pipetting error, all samples were normalized to has-miR-361-5p. The percent change in expression of miR-9 was calculated using normalized target miRNA expression in samples (2^−∆∆Ct^).

## Results

### UD-P19 and P19N Secrete Exosomes

P19N are normally cultured in a defined medium lacking fetal bovine serum (FBS) but UD-P19 are cultured in medium containing 10% FBS [18]. Since FBS contains exosomes [27], we optimized the UD-P19 culture conditions and found α-MEM with 1 × N2 supplement to be the optimal medium to culture UD-P19 in the absence of FBS. Cells cultured at 60–70% confluency in α-MEM/N2 medium for 24 h were most suitable for exosome isolation from the conditioned medium (CM) (Supplementary Figure S1). Contrary to our expectation, cells plated at a higher confluency (≥ 80%) did not release higher number of exosomes as compared to cells at 60–70% confluency. Cells plated at a low confluency (≤ 30%) released low number of exosomes. For all subsequent experiments, cells were plated at ~ 60–70% confluency to collect CM for exosome isolation.

Freshly collected CM from UD-P19 and P19N was processed immediately for exosome isolation using differential centrifugation (Supplementary Figure S2). Following removal of floating cells and microvesicles by two sequential low speed centrifugation steps, CM was ultracentrifuged to pellet down exosomes. Exosome pellets were suspended in DPBS by gentle pipetting, pooled, and ultracentrifuged to recover washed exosomes that appeared as a translucent pellet at the base of the centrifuge tube (Supplementary Figure S4). UD-P19 exosomes were sticky and difficult to suspend to a homogeneous suspension as compared to P19N exosomes. Gentle pipetting for 15–20 min was essential to obtain uniformly suspended UD-P19 exosomes. Harsh treatment such as vortexing or pipetting with force resulted in loss of exosome integrity.

**Fig. 1:**
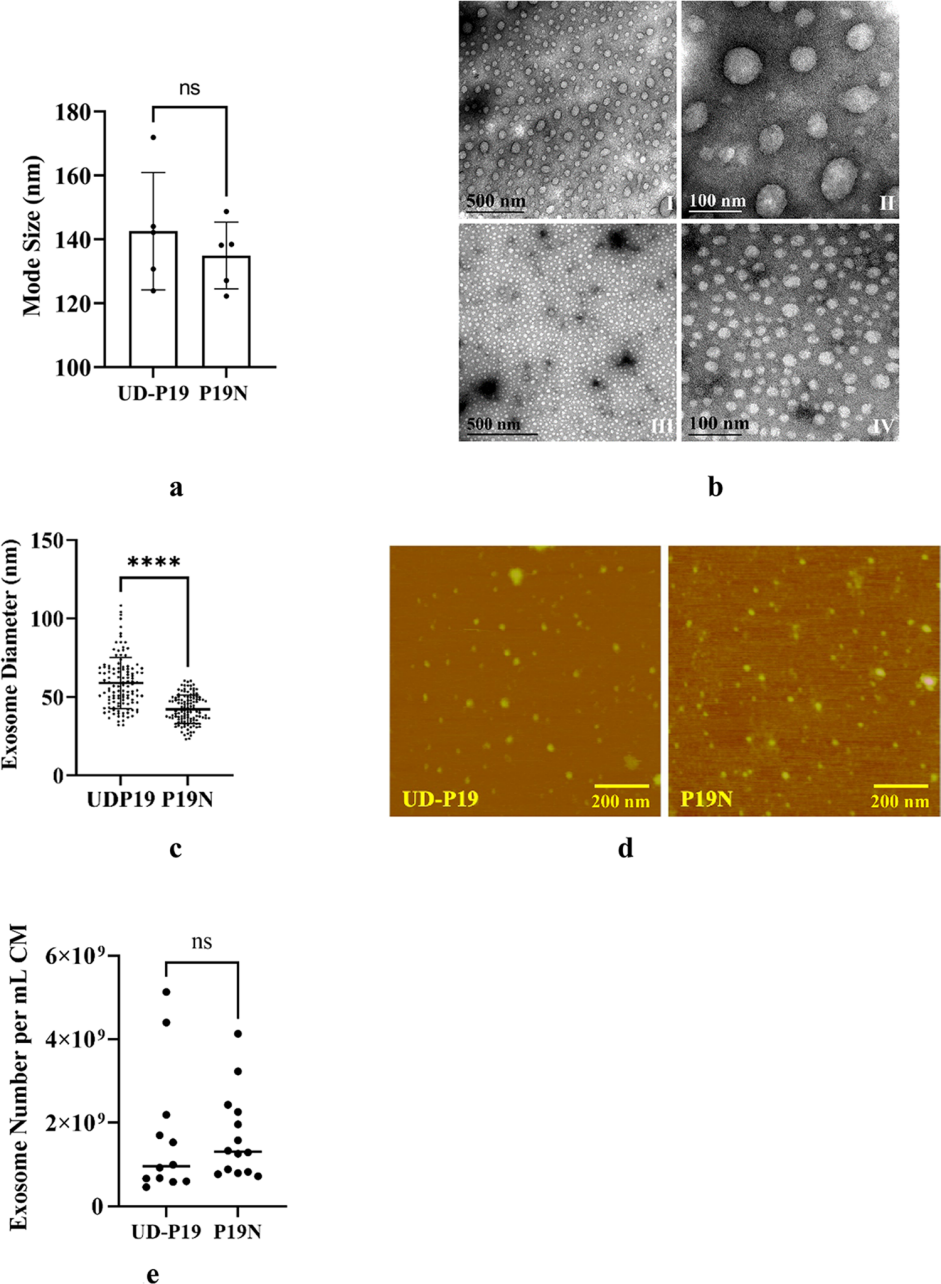
Exosome characterization. Exosomes purified by differential ultracentrifugation were processed to determine their diameter under hydrated and desiccated conditions. **1a**: The hydrodynamic mode size of UD-P19 and P19N exosomes was comparable. Statistical analysis was performed using unpaired two-tailed t-test; ns: not significant; (*n* = 5). **1b**: Representative TEM images of UD-P19 exosomes (top panel) and P19N exosomes (bottom panel) at low (scale bar = 500 nm) and high (scale bar = 100 nm) magnification. **1c**: Scatterplot showing significantly larger size of UD-P19 exosomes as compared to P19N exosomes. Statistical analysis of exosome diameter under TEM was performed using unpaired two-tailed t-test, ****p < 0.0001; (*n* = 120). **1d**: Representative tapping-mode AFM images (1 × 1 µm^2^) of UD-P19 and P19N exosomes. Scale bar = 200 nm. **1e**: UD-P19 and P19N released similar number of exosomes per volume of conditioned medium (CM). Exosome concentration was automatically calculated by NTA software. Data analysis using unpaired two-tailed t-test showed the difference in exosome number released by two cells was nonsignificant (ns; *p* = 0.9674) (*n* = 12 for UD-P19 and 14 for P19N)

UD-P19 and P19N exosome size was within the expected range (Fig. 1; Supplementary Table TS1). The hydrodynamic mode diameter determined by nanoparticle tracking analysis (NTA) was 143 nm and 135 nm for UD-P19 and P19N exosomes, respectively. Statistical analysis of data using GraphPad showed that the hydrated size of two exosomes was comparable (Fig. 1a). After depositing hydrated exosomes on transmission electron microscopy (TEM) grids and freshly-cleaved mica sheets, exosomes were dried slowly prior to their analysis on TEM and atomic force microscope (AFM), respectively. Slow drying prevented the collapse of unfixed exosomes giving them the appearance of spheres (Figs. 1b and d) and not the cup shape morphology reported in literature. Evenly distributed exosomes on TEM grid (Fig. 1b) had an average diameter of 59 nm and 42 nm for UD-P19 and P19N exosomes, respectively. Data analysis using GraphPad showed that under dry conditions, the diameter of UD-P19 exosome was significantly larger than the diameter of P19N exosomes (Fig. 1c). Based on AFM analysis, the average diameter of desiccated UD-P19 and P19N exosomes was 30 nm and 32 nm, respectively (Fig. 1d). The number of exosomes released by UD-P19 and P19N calculated using NTA were 1.65 × 10^9^ and 1.67 × 10^9^ per mL volume of conditioned medium (exosome number/one mL CM), respectively. Statistical analysis of data indicated that exosome number released by UD-P19 and P19N were not significantly different from each other (Fig. 1e).

**Fig. 2:**
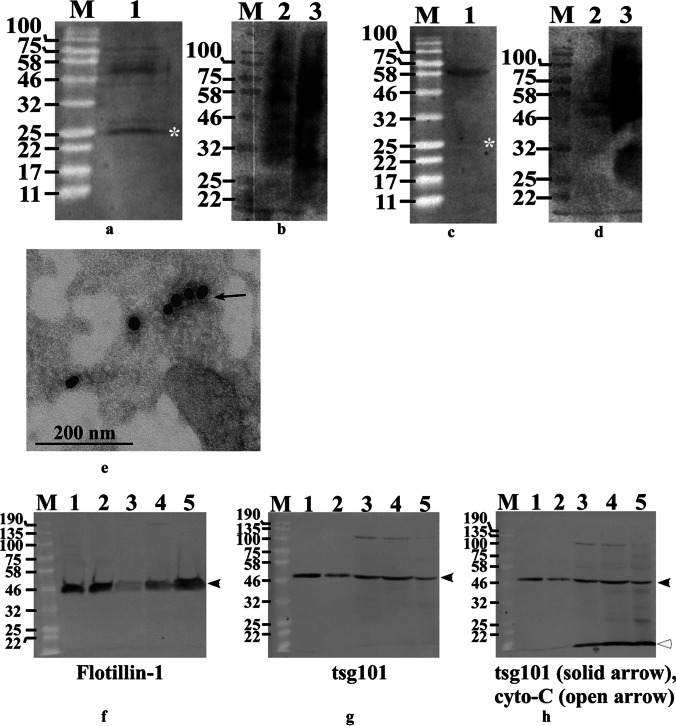
Biochemical characterization of exosomes. Presence of exosomal marker proteins in UD-P19 and P19N exosomes was determined by Western blotting and immuno-electron microscopy. Proteins from parent cell lysates and FCN lysate were used as controls. Pre-stained protein molecular weight markers (NEB) (Lane M) were included in all gels and their size is indicated in kDa on the left. **2a**: The 25 kDa non-glycosylated CD63 (white asterisk) and multiple glycosylated CD63 protein bands were detected in UD-P19 exosomes (Lane 1); **2b**: Multiple glycosylated forms of CD63 were detected in UD-P19 lysate (Lane 2: 50 µg lysate; Lane 3: 100 µg lysate). **2c**: A major glycosylated CD63 and a very faint 25 kDa CD63 (white asterisk) protein bands were present in P19N exosomes (Lane 1). **2d**: Multiple CD63 protein bands that merged to appear as a smear instead of distinct bands were seen in P19N lysate (Lane 2: 50 µg lysate; Lane 3: 100 µg lysate). **2e**: Representative electron micrograph showing surface localization of CD63 protein (arrow) on UD-P19 exosomes. Exosomes were processed for immuno-EM by indirect method using anti-CD63 and gold (18 nm) conjugated secondary antibody. Scale bar = 200 nm. **2f–2h**: Flotillin-1 (**2f**) and tsg101 (**2 g**) proteins were present in UD-P19 exosomes (Lane 1), P19N exosomes (Lane 2), and in all cell lysates (Lane 3 = UD-P19; Lane 4 = P19N; Lane 5 = FCN). Immunoreactive proteins are indicated by black arrowheads. **2h**: Membrane probed with anti-tsg101 was washed and re-probed with anti-cytochrome C. Cytochrome C protein (open arrowhead) was absent in UD-P19 exosomes (Lane 1) and P19N exosomes (Lane 2) but present in cell lysates from UD-P19 (Lane 3), P19N (Lane 4), and FCN (Lane 5)

Biochemically, exosomes were characterized by examining classical exosomal marker proteins using Western blotting. The presence and expression level of classical marker proteins is not uniform in exosomes purified from different parent cells. Therefore, we first optimized Western blot conditions in terms of exosome protein amount, lysis buffer, storage temperature for solubilized samples, and antibody dilution (Table 1, Supplementary Table TS2, Supplementary Figures S4 and S5). UD-P19 and P19N exosomes and their respective parent cells expressed CD63 although the pattern of non-glycosylated 25 kDa and glycosylated CD63 forms differed among these four samples (Fig. 2a to d). CD63 is a transmembrane protein in exosomes and its presence on the surface of UD-P19 exosomes was confirmed by immuno-electron microscopy (Fig. 2e; Supplementary Figure S6). Exosomes incubated with DPBS in lieu of anti-CD63 displayed no binding of gold conjugated secondary antibody (Supplementary Figure S6). Tetraspanin proteins, CD9 and CD81 were detected in fetal cortical neuronal lysate (positive control) (Supplementary Figure S5) but not in UD-P19 and P19N exosomes. Membrane associated exosome marker protein, flotillin-1, and cytosolic protein associated with multivesicular bodies, tsg101, were present in UD-P19 and P19N exosomes, their respective parent cells, and FCN (Figs. 2f and g). Cytochrome C, an intracellular protein associated with mitochondria, was absent in UD-P19 and P19N exosomes but present in all three cell lysates (Fig. 2h). These data authenticated the purification of UD-P19 and P19N exosomes from their respective CM.

### UD-P19 and P19N Internalized P19N Exosomes

**Fig. 3:**
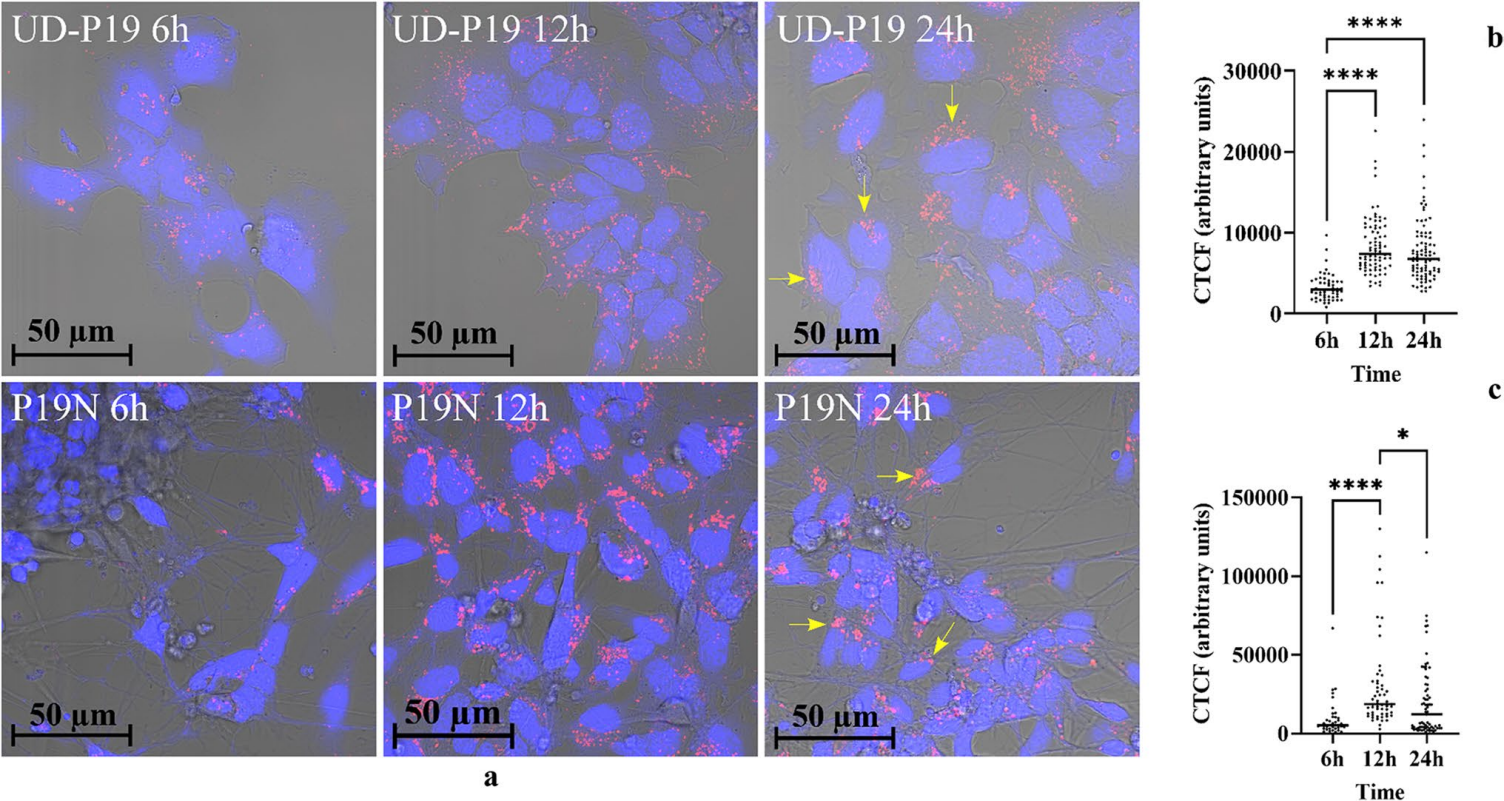
Time-course of P19N exosome internalization. **3a**: Representative images of UD-P19 (upper panel) and P19N (lower panel) showing a time course of internalized Dil-labeled P19N exosomes. Cells were incubated in 350 µL medium with 40 µg/mL Dil-labeled exosome concentration (red fluorescence). Control cells received 350 µL of Dil/DPBS processed in the same way as exosomes and incubated for 24 h time duration only. Cultures terminated at 0, 6, 12, and 24 h were fixed and counterstained with DAPI (blue). Images were captured using an EC Plan-Neofluar 40x/1.30 NA objective on a confocal microscope (Zeiss, LSM-700). Exosome enrichment in the nuclear region of cells is indicated by yellow arrows. Scale bar = 50 µm. **3b**: Scatterplot showing a time course of Dil-fluorescence intensity in UD-P19. Dil-fluorescence intensity was measured in 50–100 UD-P19 at each time point using ImageJ and quantitated by calculating CTCF (see methods for details). Statistical analysis of CTCF was performed using ordinary one-way ANOVA. The pairwise comparison among different time points was performed by Tukey’s multiple comparisons test. 6 h vs 12 h: ****p < 0.0001; 6 h vs 24 h: ****p < 0.0001; 12 h vs 24 h *p* = 0.5249). **3c**: Scatterplot showing a time course of Dil-fluorescence intensity in P19N. Dil-fluorescence intensity was measured in 50–100 individual P19N at each time point using ImageJ and quantitated as in 3b. Statistical analysis of CTCF was performed using ordinary one-way ANOVA and pairwise comparison among different time points was performed by Tukey’s multiple comparisons test. 6 h vs 12 h: ****p < 0.0001; 6 h vs 24 h: *p* = 0.0527; 12 h vs 24 h: *p = 0.0339)

Internalization of exosomes is the key step to elicit responses in recipient cells. Hence, we examined uptake of Dil-labeled P19N exosome by UD-P19 and P19N. Dil is a fluorescent lipophilic membrane dye that did not affect the size of exosomes. The average hydrodynamic mode diameter of Dil-labeled exosomes was ~ 129 nm. Both UD-P19 and P19N internalized P19N exosomes in a concentration-dependent (data not shown) and time-dependent manner (Fig. 3). Presence of internalized exosomes within cells was confirmed by optical slicing (z-stacks) using a Zeiss confocal microscope (Supplementary Figures S7a and b). Control cultures were incubated with Dil/DPBS processed in the same manner as Dil/exosomes. No red fluorescence was observed in control cells, indicating lack of Dil micelle formation during Dil-exosome labeling procedure and their uptake by cells (Supplementary Figures S7c and d).

Exosome uptake was higher at a 40 µg/mL than at 20 µg/mL exosome concentration (data not shown). A follow-up study to determine time-dependent exosome internalization was carried out using 40 µg/mL exosome concentration. Cells incubated with Dil-exosomes were terminated at 0 h, 6 h, 12 h, and 24 h and cell images were captured at each time-point using Zeiss confocal microscope (Fig. 3a). Intensity of fluorescence emitted by internalized Dil-exosomes was quantified using ImageJ software and expressed as corrected total cell fluorescence (CTCF). Maximum internalization was observed at 12 h in both cells. At 24 h, the CTCF in UD-P19 decreased as compared to 12 h but remained significantly higher than 6 h time point (Fig. 3b). In contrast, the CTCF was significantly reduced in P19N at 24 h as compared to 12 h time point (Fig. 3c) and this could reflect rapid assimilation of exosomes in P19N. Notably, internalized exosomes were localized around nuclei at 24 h time point in both cells (yellow arrows in Fig. 3a).

### P19N Exosomes Exerted Biological Effects on UD-P19

**Fig. 4:**
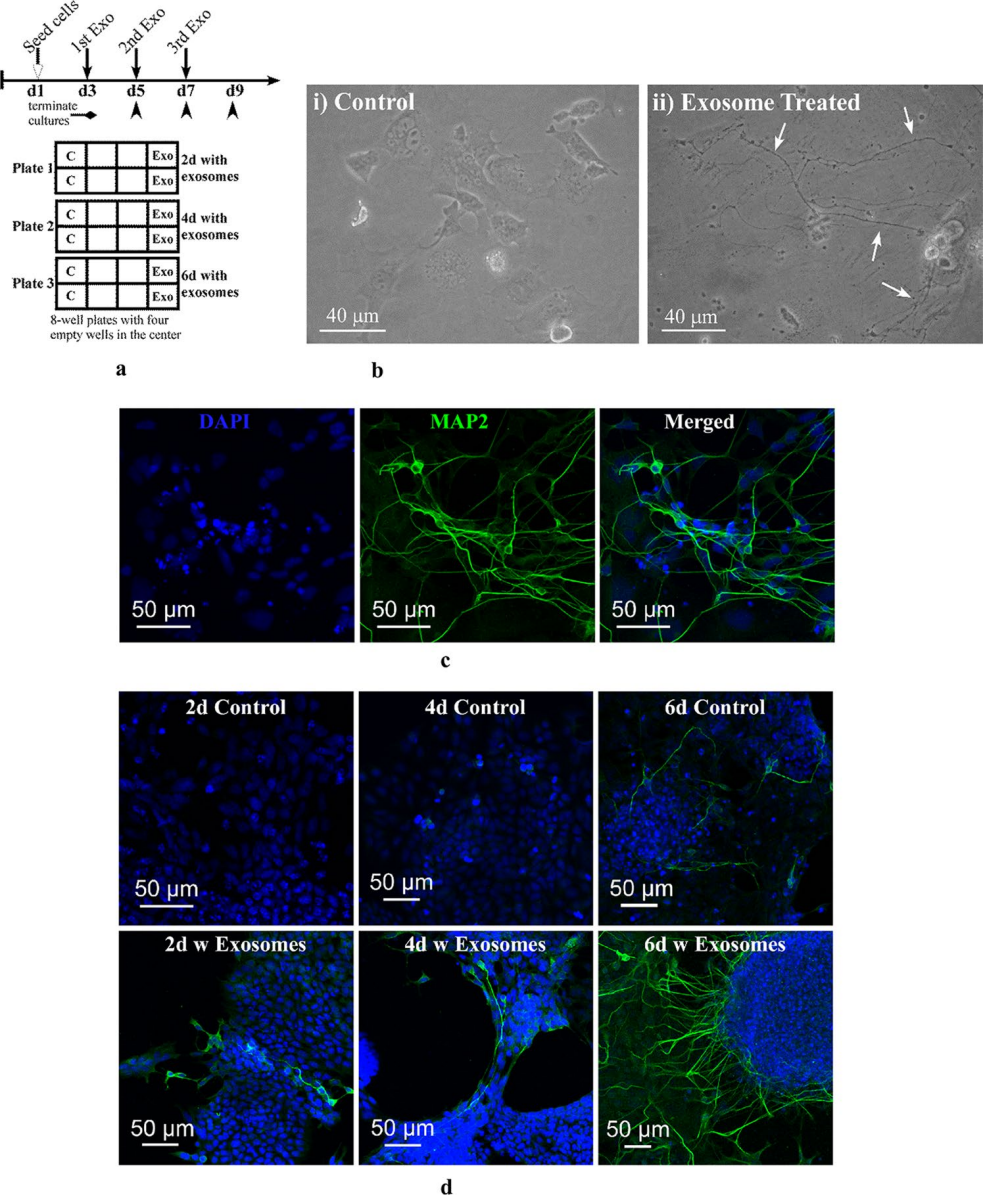

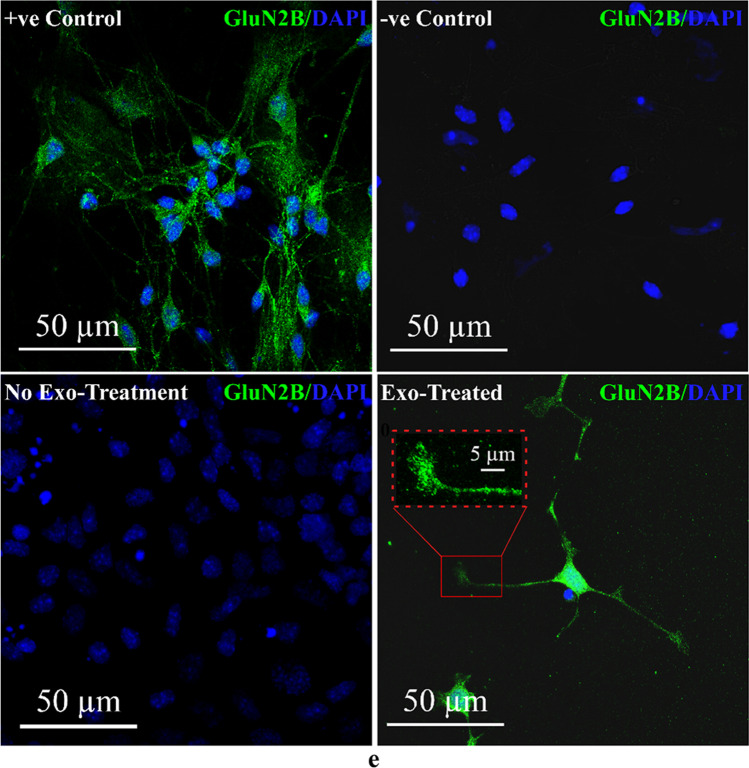
Biological effects of P19N exosomes on UD-P19. **4a**: Schematic paradigm of the exosome treatment of UD-P19. Cells were cultured in the presence of P19N exosomes [350 µL of 40 µg/mL exosome] with change of medium containing freshly isolated exosomes every 2d (downward arrows). Cultures terminated after 2, 4, and 6d of exosome exposure (upward arrowheads) were processed for phase contrast imaging. **4b**: Representative phase contrast images of UD-P19 with and without 6d exposure to P19N exosomes. **4bi**: Untreated cells (controls); **4bii**: Incubation with P19N exosomes for 6d induced differentiation of UD-P19 to neurons with long neurites (white arrows). Images were captured using 20 × phase objective on a Nikon Eclipse TS100 inverted microscope. Scale bar = 40 µm. **4c–4e**: UD-P19 exposed to P19N exosomes for 2, 4, and 6d were processed for MAP2- and 6d exposed for GluN2B- immunostaining by indirect method. Nuclei were counterstained with DAPI (blue). Scale bar = 50 µm. **4c**: Representative confocal image of UD-P19 exposed to exosomes for 6d. UD-P19 differentiated into neurons display MAP2 immunostaining (green fluorescence). **4d**: Representative confocal images showing time-dependent effect of exosomes on UD-P19 differentiation into neurons. Untreated UD-P19 showed some MAP2 positive cells (upper panel). MAP2 positive cells with long neurites increased in number with increase in exosome exposure time from 2 to 6d (lower panel). **4e**: Representative confocal images of GluN2B-positive neurons differentiated following 6d exosome exposure. The 8d old RA-induced P19 neurons served as positive control (+ ve Control) for GluN2B immunostaining (green fluorescence) (Top Panel). The 8d old RA-induced P19 neurons incubated with BSA in lieu of primary antibody served as negative control (-ve Control) and lacked green fluorescence in cells (Top Panel). UD-P19 cultured in the absence of exosomes lacked GluN2B expression as they had no green fluorescence (No Exo-Treatment) (Lower Panel). UD-P19 cultured for six days with P19N exosomes showed punctate GluN2B immunostaining in neurites (Exo Treated) (Lower Panel). A single exosome-induced neuron displaying GluN2B staining in soma and neurites (Exo-treated) is shown here. Inset (red dotted line rectangle) shows punctate GluN2B immunostaining at higher magnification (Scale bar = 5 µm). All cells were counterstained with nuclear stain, DAPI. Scale bar = 50 µm

P19N exosomes uptake by UD-P19 prompted us to examine impact of P19N exosomes on UD-P19. We cultured UD-P19 with P19N exosomes for six days with fresh exosomes containing medium fed every two days. Cultures were terminated after 2, 4, and 6 days of exosome exposure (Fig. 4a). Remarkably, exosome exposure of UD-P19 recapitulated RA mediated differentiation of UD-P19 to P19 neurons. Exosome exposure induced formation of embryoid bodies (EBs) with the exception that exosome-induced EBs were smaller and remained attached to the surface (red arrowheads in Supplementary Figures S8a, b, and c) unlike free-floating RA-induced EBs (data not shown here). Notably, six days exosome exposure induced morphological transformation of UD-P19 to cells with long neurite-like processes (Fig. 4bii). Control UD-P19 cultured simultaneously in the absence of exosomes retained their flat cell appearance (Fig. 4bi).

Development of neurites requires assembly of microtubules, a process dependent upon interaction with MAP2 and other proteins [28]. We reasoned if the observed long neuron like processes were true neurites then they should be positive for MAP2. Therefore, we initially used six days exosome-exposed cells to examine MAP2 expression by immunocytochemistry. A large number of cells showed robust MAP2 expression (Fig. 4c). A follow-up study examining MAP2 positive cells highlighted a time-dependent effect of P19N exosomes in inducing differentiation of UD-P19 to neurons. An increase in MAP2-positive cells was observed with increase in exosome exposure time thus reflecting time-dependent biological effects of P19N exosomes on morphological differentiation of UD-P19 to neurons (Fig. 4d: lower panel; Supplementary Figure S8). Untreated control cultures did not form EBs and displayed few MAP2-positive cells only after six days of culture (Fig. 4d: upper panel; Supplementary Figure S8). Neurite growth during embryonic development and neuronal plasticity are regulated by NMDA receptor subunit, GluN2B [29, 30]. We therefore examined expression of GluN2B subunit in exosomes-induced neurons. Exosome-induced neurons expressed GluN2B subunit. Cells with neuron-like morphology that developed in UD-P19 cultured in the absence of exosomes (control cultures) did not express GluN2B subunit (Fig. 4e). Thus, GluN2B immunostaining allowed us to distinguish exosome-mediated cellular differentiation of UD-P19 to P19 neurons from morphological differentiation of UD-P19 cultured in the absence of exosomes.

**Fig. 5:**
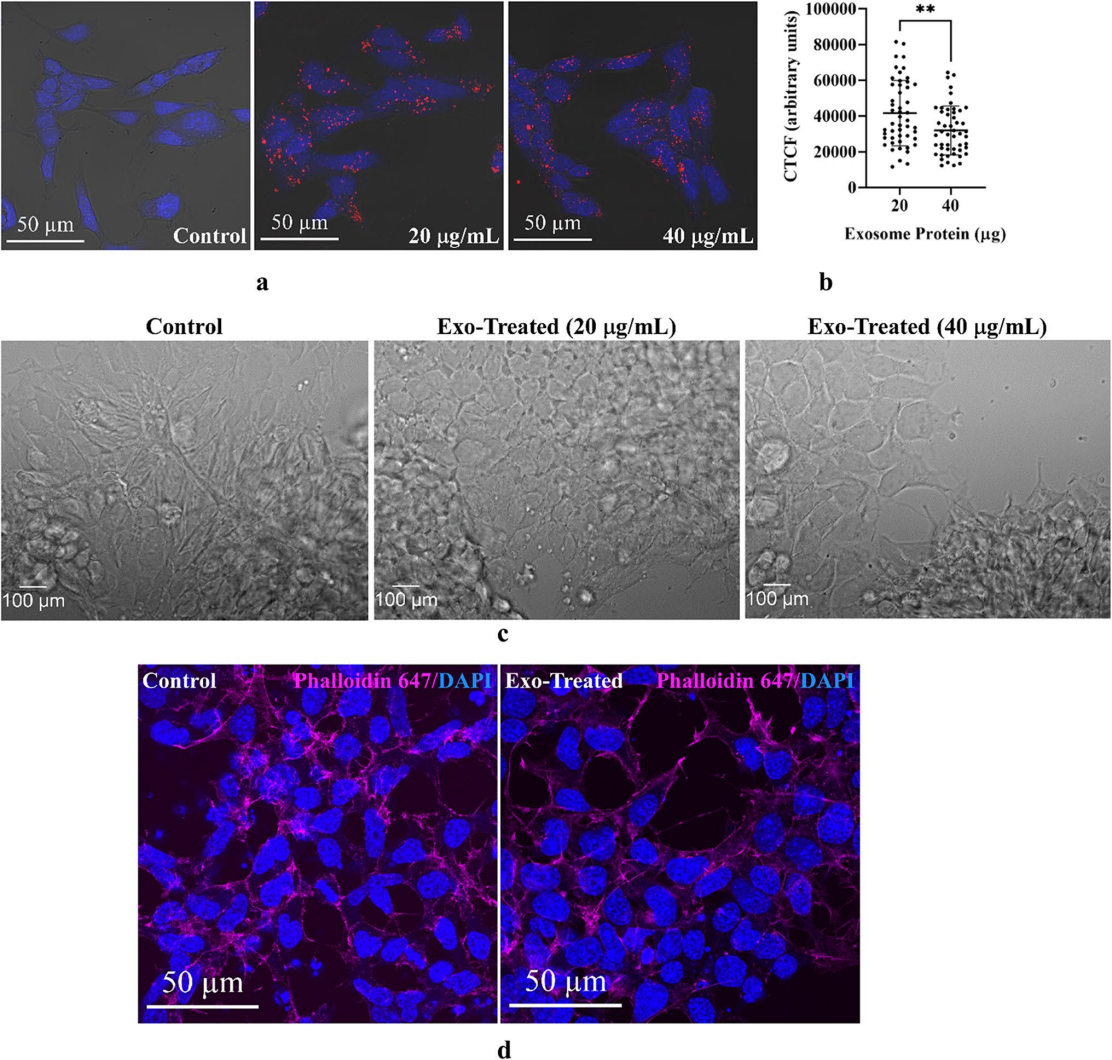
Effect of UD-P19 exosomes on UD-P19. **5a**: Representative images of UD-P19 showing concentration-dependent internalization of UD-P19 exosomes. UD-P19 were incubated independently with 20 µg/mL and 40 µg/mL of Dil-labeled UD-P19 exosomes (red fluorescence) for 24 h. Control cells received 350 µL of Dil/DPBS processed in the same way as exosomes and cultured for 24 h. Cultures terminated at 24 h were fixed and counterstained with DAPI (blue). Images captured using an EC Plan-Neofluar 40x/1.30 NA objective on a confocal microscope (Zeiss, LSM-700) showed uptake of UD-P19 exosomes but no enrichment of internalized exosomes in the nuclear region of cells irrespective of the exosome concentration. Scale bar = 50 µm. **5b**: Scatterplot showing UD-P19 exosome concentration-dependent Dil-fluorescence intensity in UD-P19. Dil-fluorescence intensity was measured in 50 cells per exosome concentration using ImageJ and quantitated by calculating CTCF (see methods for details). Statistical analysis of CTCF between two exosomes concentrations was performed using unpaired two-tailed t test. 20 vs 40 µg: **p < 0.0035. **5c**: Representative DIC images of control and exosome-treated UD-P19. The cell morphology was similar in control UD-P19 and UD-P19 incubated with two different concentrations of UD-P19 exosomes for 6d. Scale bar = 100 µm. **5d**: Representative confocal images of control and exosome-treated UD-P19 (40 µg/mL) stained with Alexa Fluor™ 647 Phalloidin (bright pink) and DAPI (blue). The bright pink fluorescence shows actin cytoskeleton of UD-P19 and blue fluorescence highlights the nuclei. Scale bar = 50 µm

To confirm P19N exosome-mediated cellular differentiation of UD-P19 to P19N, we incubated UD-P19 with UD-P19 exosomes using cell culture paradigm in Fig. 4a. Prior to performing exosome exposure study, we determined internalization of UD-P19 exosomes by UD-P19. UD-P19 were incubated with two concentrations of Dil-labeled UD-P19 exosomes for 24 h. Images were captured using Zeiss confocal microscope as in Fig. 3. Analysis of images showed maximum internalization of UD-P19 exosomes by UD-P19 was at 20 µg/mL exosome concentration (Fig. 5a and b). Control cells had no red fluorescence (not shown here). Internalized exosomes were somewhat uniformly distributed in cells unlike P19N exosomes that accumulated in the perinuclear region. Next, we examined effect of UD-P19 exosomes on UD-P19 by culturing UD-P19 for 6 days with 20 µg/mL and 40 µg/mL UD-P19 exosome concentrations. Exosome treated cells received freshly isolated exosomes every 48 h. Control cultures received fresh medium every 48 h at the same time as the exosome treated cells. The 40 µg/mL exosome concentration was used to obtain data comparable to UD-P19 exposed to 40 µg/mL P19N exosomes (Fig. 4c). At the end of the six days, cultures were terminated and differential interference contrast (DIC) images of fixed cells were captured under Leica DMI6000 B inverted microscope. No difference in the morphology of control and exosome-treated UD-P19 was noted (Fig. 5c). To confirm that UD-P19 exosomes did not exert morphological differentiation effects on UD-P19, we plated UD-P19 at a low density and cultured them for six days with UD-P19 exosomes as in Fig. 5c. At the end of six days, cells were stained for cytoskeletal protein, F-actin with phalloidin 647 and counterstained with DAPI. Cell images were captured using Zeiss confocal microscope. No difference in cell morphology between control and exosomes-treated UD-P19 was evident based upon F-acting staining (Fig. 5d). Collectively, these data strongly argue in support of P19N exosomes-mediated cellular differentiation of UD-P19 to neurons.

### P19N Exosomes Contain Pro-neurogenic Non-coding RNAs

**Fig. 6:**
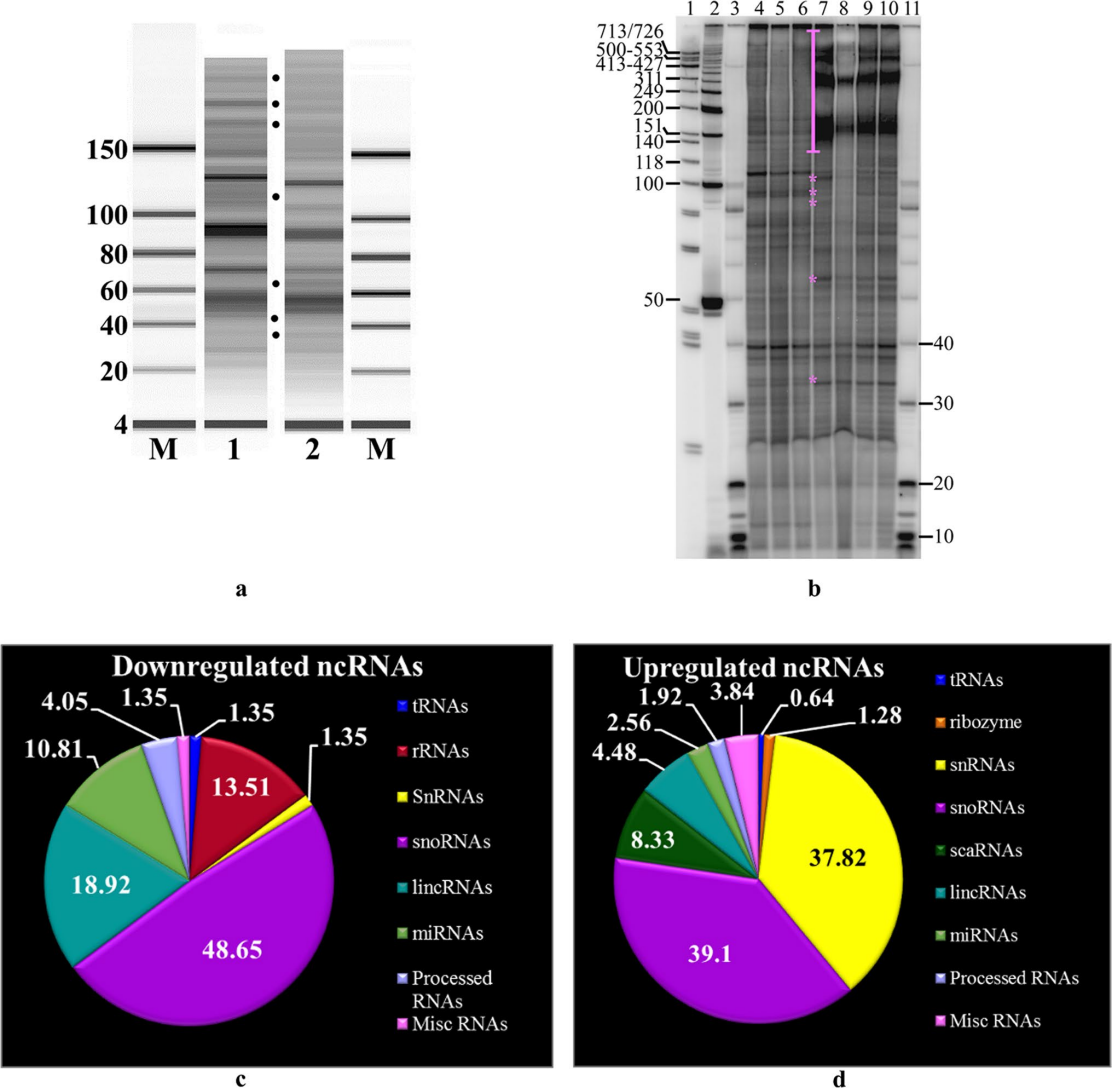

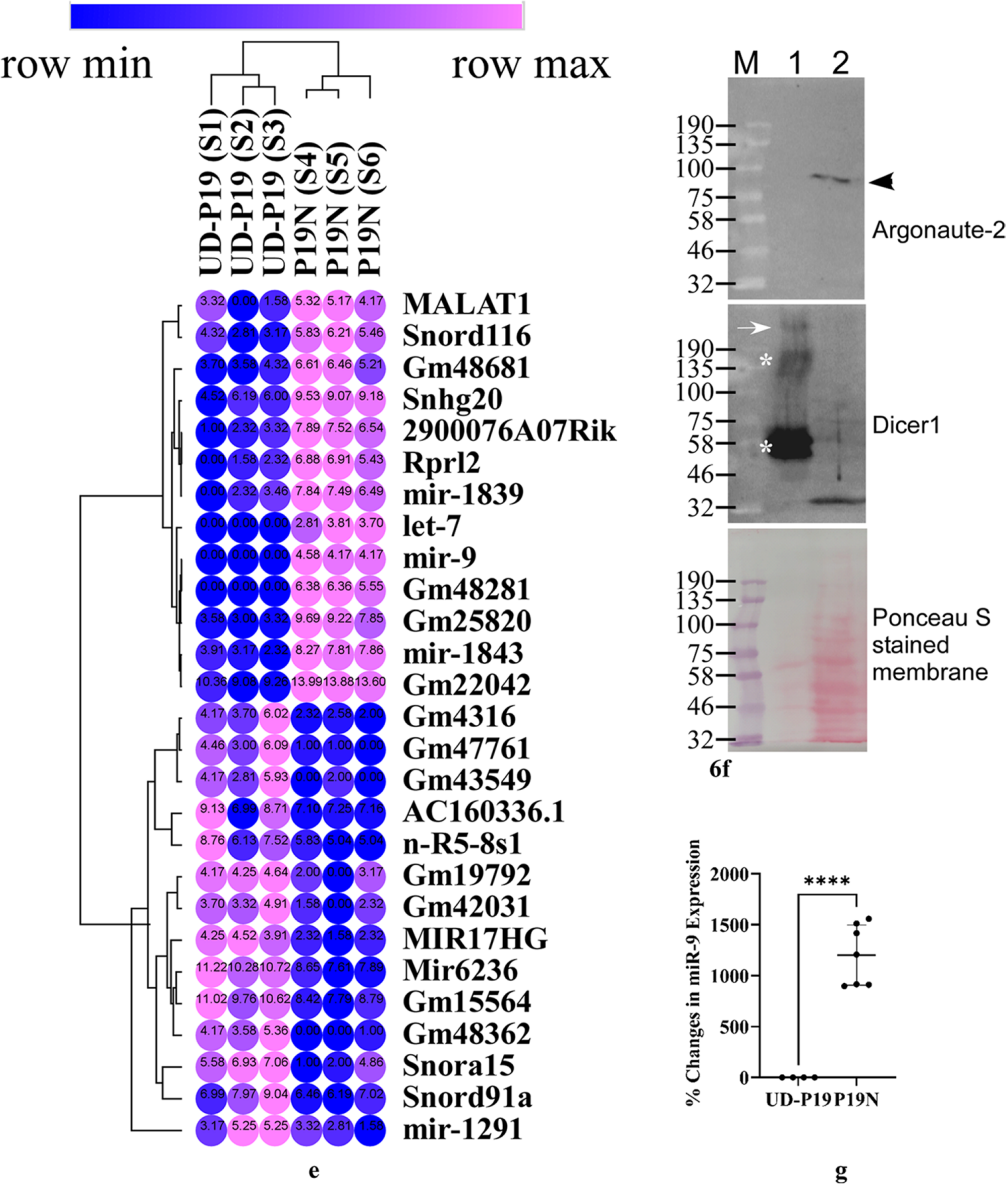
RNA cargo of UD-P19 and P19N exosomes. **6a**: A representative gel image of UD-P19 (Lane 1) and P19N (Lane 2) exosomal RNAs separated on a BioAnalyzer chip. Black dots highlight differentially present exosomal RNAs. Lanes M: Small RNA ladder with molecular size (nucleotides) indicated on the left. **6b**: Autoradiograph showing size of UD-P19 and P19N exosomal RNAs. Radiolabeled exosomal RNAs separated on denaturing sequencing gel with ^32^P-end-labeled DNA molecular weight markers were analyzed on PhosphorImager. Salient differences in the ± of RNAs are indicated by pink asterisks and pink vertical line. Lane 1: ^32^P-labeled ΦX174 DNA/HinfI DNA molecular weight markers with molecular size (nucleotides) indicated on the left; Lane 2: ^32^P-labeled 50 bp DNA molecular weight ladder; Lanes 3 and 11: ^32^P-labeled 10 bp DNA molecular weight ladder with molecular size (nucleotides) indicated on the right; Lanes 4–6: UD-P19 exosomal RNAs from three biological replicates; Lanes 7–10: P19N exosomal RNAs from four biological replicates. **6c** and **6d**: Pie charts showing ncRNAs downregulated (%) and enriched (%) in P19N exosomes as compared to UD-P19 exosomes, respectively. **6e**: Two-way hierarchical clustering of salient transcripts. The heat map was generated using the log_2_ transformed expression values of exosomal ncRNAs from three biological replicates per parent cell (indicated above the columns). Each row of circles represents a single ncRNA. Pink circles denote upregulated ncRNAs while blue denotes downregulated ncRNAs. The values in the heat map are mapped to colors using the minimum and maximum of each row independently. The numbers in the circles represent the log_2_ transformed expression values. **6f**: Western blot analysis to detect argonaute-2 protein (arrowhead on the right) and Dicer1 protein (full length indicated by white arrow and degraded Dicer-1 by white asterisks on the left) in P19 exosomes (Lane 1) and P19N lysate (Lane 2). Lane M: Pre-stained protein molecular weight markers (NEB) with molecular weight (kDa) indicated on the left. **6 g**: Scatter plot showing normalized levels of miR-9 in UD-P19 and P19N exosomes. RNAs extracted from UD-P19 and P19N exosomes were determined by RT-qPCR using hsa-miR-361-5p as housekeeping control. Statistical analysis was performed using unpaired two-tailed t-test, ****p < 0.0001

Non-coding RNAs (ncRNAs) are implicated in stem cell-derived neurogenesis during development [31]. Because exosomes induced differentiation of UD-P19 to neurons, we reasoned that comparing small RNA transcriptomes of UD-P19 and P19N exosomes might allow us to identify candidate ncRNAs potentially involved in neuronal differentiation. Three biological replicates of UD-P19 exosomes and three biological replicates of P19N exosomes (total six independent exosome preparations) were processed to isolate their RNA. All six exosomal RNA samples were analyzed on BioAnalyzer, by ^32^P-end-labeling, and lastly, by small RNA-seq. Initial analysis of exosomal RNAs on a BioAnalyzer using small RNA chips (Supplementary Figure S9) identified some differences in RNAs between UD-P19 and P19N exosomes (Fig. 6a: black dots). A higher resolution of exosomal RNAs by end-labeling RNAs with ^32^P-γ-ATP and separation on a denaturing polyacrylamide gel identified a range of small and high molecular weight RNAs in UD-P19 and P19N exosomes (Fig. 6b). Comparison of ^32^P-RNAs identified several similar sized RNAs common to both exosomes. Differences in RNAs quantity and the presence of novel RNAs specific to UD-P19 or P19N exosomes were noted based on intensity of radiolabeled RNA bands (Fig. 6b: pink asterisks and pink vertical line).

The identity of unique and common exosomal RNAs was determined by small RNA-Seq of double stranded barcoded cDNA libraries generated using 200 ng exosomal RNA per sample. The quality of cDNA libraries was assessed (Supplementary Figure S10) prior to their quantification and pooling in equimolar amount for sequencing on an Illumina NextSeq 500 platform. FASTQ files generated from the raw data were processed independently using the default parameters of CLC Genomics Workbench to generate paired sequences that were combined by concatenation. The number of reads in exosomes from UD-P19 and P19N ranged between ~ 1.6 to ~ 4 million with an average of ~ 2.6 million reads per sample. Exosomal transcripts annotated using *Mus musculus* GRCm38.ncRNA identified 69,410 to 100,115 ncRNAs with a mean of 94,320 (data can be accessed at GEO Series accession number GSE152655) (Supplementary Tables TS3 and TS4). Of these, 5,880 ncRNAs were differentially associated with UD-P19 and P19N exosomes. Next, we applied three filtering criteria, namely, FDR ≤ 0.05, Ensembl stable genes, and removal of low expressing genes to identify differentially enriched exosomal ncRNAs (Supplementary Figure S11). Out of 5,880 ncRNAs, 233 ncRNAs met these filtering criteria (Supplementary Table TS5). Among the 233 ncRNAs, 74 ncRNAs had lower expression and 159 ncRNAs had higher expression in P19N exosomes relative to the UD-P19 exosomes (Table 2). A GO:TERM analysis of the differentially expressed exosomal transcripts highlighted differential enrichment of several functional classes of transcripts related to neuron differentiation, regulation of synapse organization and long-term synaptic potentiation, regulation of gene expression, negative regulation of apoptotic process, and miRNA-mediated gene silencing among others (Table 3; the complete list in Supplementary Table TS6). Most importantly, Gene Ontology (GO) term analyses using Mouse Genome Informatics identified some crucial molecular function terms in neurogenesis such as BMP and Wnt signaling pathways, tyrosine phosphorylation of STAT protein, and cellular response to leukemia inhibitory factor that were associated with P19N exosomes’ transcripts (Table 3, Supplementary Table TS6).

**Table 2:**
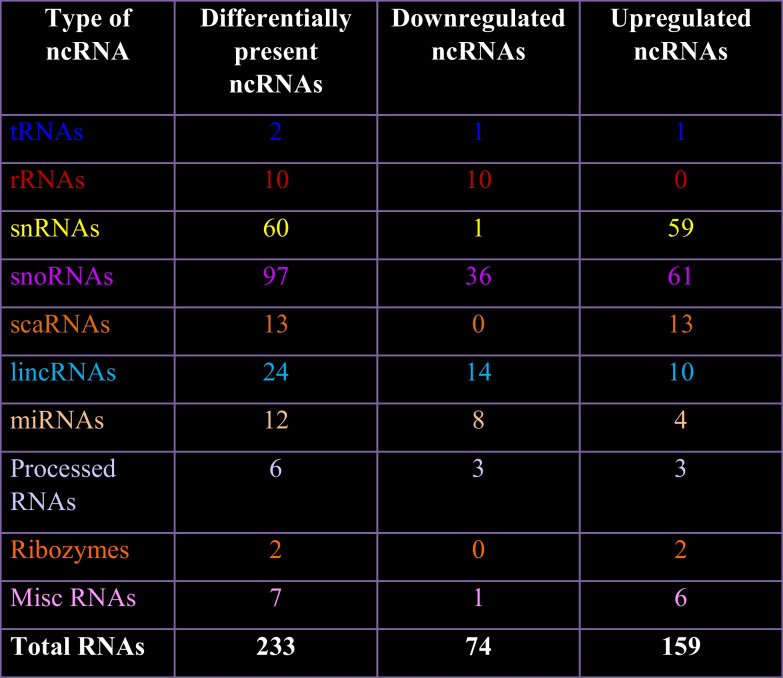
Differentially enriched ncRNA types in P19N exosomes relative to the UD-P19 exosomes

**Table 3:** A partial list of MGI GO TERMs identified that were associated with P19N exosome transcripts

Biological domain	GO ID	GO Term
Development and differentiation	GO:0,021,522 GO:0,030,424 GO:0,050,807 GO:0,060,291	spinal cord motor neuron differentiation, axon, basal dendrite, regulation of synapse organization, long-term synaptic potentiation
Gene expression	GO:0,035,195 GO:0,035,195 GO:0,010,629 GO:0,010,628 GO:0,016,442 GO:0,000,381 GO:0,035,278 GO:0,016,442 GO:0,006,402 GO:0,010,468 GO:0,005,732	regulation of gene expression,, regulation of alternative mRNA splicing, via spliceosome, negative regulation of gene expression, miRNA binding, miRNA-mediated gene silencing, miRNA-mediated gene silencing by inhibition of translation, mRNA catabolic process, rRNA 2'-O-ribose methylation guide activity, negative regulation of gene expression, positive regulation of gene expression, sno(s)RNA-containing ribonucleoprotein complex, RISC complex,
Cellular response	GO:0,071,241 GO:0,071,230 GO:0,070,482 GO:1,990,830	glucose metabolic process, cellular response to inorganic substance, cellular response to amino acid stimulus, cellular response to leukemia inhibitory factor, response to oxygen levels, reactive oxygen species metabolic process
Signaling pathways	GO:0,030,509 GO:0,016,055	BMP signaling pathway, Wnt signaling pathway, tyrosine phosphorylation of STAT protein

We manually grouped differentially expressed exosomal ncRNAs according to their class, their percent distribution, and absence/presence in P19N exosomes relative to UD-P19 exosomes (Table 2 and Fig. 6c and d). The two-way hierarchical clustering map of exosomal transcripts generated using the log_2_-transformed expression values depicted distinct enrichment or depletion of transcripts in UD-P19 and P19N exosomes (Fig. 6e; complete heat map in Supplementary Figure S12). All differentially present rRNAs were depleted in P19N exosomes. All differentially enriched snRNAs, except one, were upregulated in P19N exosomes. LincRNAs such as Snhg20, AC160336.1, 2900076A07Rik, Gm48681, Gm48281, Malat1, Gm31600 and 2810429I04Rik were particularly enriched in P19N exosomes while Mir17hg, Gm12840, Gm42047, Gm20186, and Gm4316 were enriched in UD-P19 exosomes (Fig. 6e; Supplementary Figure S12). Notable examples of ncRNAs that were preferentially enriched in P19N exosomes were mir-9, let-7 (microRNAs), Malat1 (lincRNA) and preferentially depleted in P19N exosomes were miR-6236 (microRNA), Mir17hg and GAS5 (lincRNAs) and sngh1 (processed RNAs) (Fig. 6e, Supplementary Table TS5). Small RNA-seq data analysis revealed the presence of pro-neurogenic ncRNAs/miRNAs in P19N exosomes such as let-7 and Malat-1 suggesting that these could potentially mediate the biological effects of P19N exosomes on UD-P19. UD-P19 exosomes contained pro-stemness ncRNAs/miRNAs such as GAS5 that could potentially help maintain stemness of UD-P19.

Several ncRNAs/microRNAs in P19N exosomes were present as pre-miRNAs. Unprocessed exosomal ncRNAs/microRNAs can only be effective in regulating gene expression of recipient cells if they undergo maturation process through cleavage by the RNase III enzyme, Dicer [32]. Western blot analysis detected Dicer1 protein but not argonaute-2 protein in P19N exosomes (Fig. 6f). Argonaute-2 forms the RNA-induced silencing complex (RISC) in the cytoplasm.

Since miR-9 is a versatile regulator of neurogenesis, we validated its association with P19N exosomes by real-time PCR using the housekeeping reference, hsa-miR-361-5p to normalize miR-9 levels. The normalized qPCR data showed significant expression of miR-9 in P19N exosomes and absence in UD-P19 exosomes (Fig. 6g) thus confirming small RNA-Seq results that identified exclusive presence of miR-9 in P19N exosomes (Figs. 6e).

## Discussion

Pluripotent P19 cells offer an excellent model system to study neurodevelopment [16, 33, 34]. In the present study, we used these cells to obtain evidence for neuronal exosomes-mediated neurogenesis of pluripotent P19 cells. Our data demonstrate that UD-P19 and P19N released exosomes with classic exosome characteristics. Both UD-P19 and P19N internalized P19N exosomes. Long-term (6 days) exposure to P19N exosomes and not UD-P19 exosomes induced cellular differentiation of UD-P19 to MAP2- and GluN2B-positive neurons. Exosome-mediated neuronal differentiation of UD-P19 was exosome exposure time dependent. P19N exosomes contained pro-neurogenic ncRNAs. UD-P19 exosomes were enriched with ncRNAs that support maintenance of stem cell characteristics and stem cell proliferation.

Exosomes originate in multivesicular bodies of cells and their size ranges between ~ 40 and 150 nm [4, 5]. In the present study, exosomes from CM of UD-P19 and P19N were subjected to their size measurement under hydrated and dry conditions. The hydrodynamic mode size was similar for UD-P19 and P19N exosomes and it was two to three times larger than their dry size. This difference was expected as loss of fluid will cause exosomes to shrink in size. The dry size determined by TEM showed UD-P19 exosomes to be significantly larger than P19N exosomes. These results agree with ultrastructural studies reporting neuronal exosome size to range between 40 and 80 nm [12]. Neuronal exosomes size ranges between 60–70 nm when still present within the multivesicular bodies of neuronal soma [35]. The significance of exosome size difference between two parent cells is not known and requires additional studies. Under TEM, UD-P19 and P19N exosomes appeared as ball-like structures. We attribute the ball-like morphology to slow dehydration of ‘unfixed’ exosomes that results in uniform shrinkage of exosomes. The cup-shaped morphology often reported in the literature for fixed exosomes is an artifact [36]. The dry size determined by AFM showed the size of UD-P19 and P19N exosomes was quite similar, and smaller than that obtained using TEM. This could be explained by loss of loosely adhering large sized exosomes during our wash step used for removing salt crystals from the mica surface. The surface of mica is hydrophilic with a negative charge in aqueous solution and exosomes are also negatively charged [37]. Thus, exosomes may not strongly adsorb onto the mica surface owing to electrostatic repulsion in contrast to the carbon surface used for TEM, as shown by the lower exosome density in Fig. 1d.

The physiological status of parent cells dictates the number of exosomes released. UD-P19 are flat cells and can release exosomes from the entire surface. Neurons are polarized cells with soma and neuronal processes. Neurons tend to release exosomes from cell body or the dendrite [38]. Despite the difference in cell shape, UD-P19 and P19N released similar number of exosomes when expressed per mL CM (exosome number/CM volume). Both UD-P19 and P19N exosomes expressed common exosome marker proteins, CD63, flotillin-1, and tsg101. CD63, a transmembrane protein, was present on the surface of UD-P19 exosomes. The cytosolic protein, cytochrome C and CD9 and CD81 were absent in UD-P19 and P19N exosomes. CD9 and CD81 are normally present in ectosomes and absent in exosomes [39–41]. In contrast, CD63 is enriched in exosomes [41, 42]. Absence of CD9 and CD81 in UD-P19 and P19N exosomes suggests that our exosome isolation procedure utilizing differential centrifugation allowed exosome isolation without ectosome contamination. Together, these data confirmed purification of exosomes enriched fractions from CM of UD-P19 and P19N.

Intercellular communication through exosomes can regulate stem cell differentiation [43]. Since UD-P19 can potentially be classified as “pluripotent stem cells”, P19N exosomes could stimulate UD-P19 differentiation to neurons. When incubated with Dil-labeled P19N exosomes, both UD-P19 and P19N internalized exosomes in a concentration and time dependent manner albeit with some differences in the kinetics. Maximum exosome uptake was seen at 12 h and thereafter decline in internalized exosomes was faster in P19N but not in UD-P19. The CTCF of internalized exosomes in UD-P19 was similar at 12 h and 24 h time points. At the 24 h time point, exosomes accumulated in the perinuclear region of both cells. The perinuclear region is rich with endoplasmic reticulum, nucleoplasm reticulum, and late endosomes and thus, it is likely that internalized exosomes release their bioactive cargo in this location to effectively modulate the gene expression of recipient cells [44–46]. Accumulation of exosomes in the perinuclear region led us to examine the impact of long-term incubation of UD-P19 with P19N exosomes. Exosome exposure for six days recapitulated RA-mediated cellular differentiation events from formation of embryoid bodies to differentiation of neurons with two exceptions. One, exosomes induced EB formation. These EBs were small sized. Second, exosome-induced EBs remained attached to culture dishes. Differentiation of UD-P19 into neurons was observed within two days of exosome exposure. However, the number of differentiating neurons increased from two to six days of exosome exposure and this effect on neuronal differentiation and neuritogenesis was readily evident with MAP2 staining. Control cells cultured in the absence of exosomes for 6 days were occasional MAP2-positive but GluN2B negative suggesting their morphological differentiation. In contrast, neurons differentiated following exposure of UD-P19 to P19N exosomes for 6 days were MAP2 and GluN2B positive confirming their cellular differentiation. One of the subunits of excitatory NMDA receptors, GluN2B is important in neurite growth during development [29]. Cellular differentiation of UD-P19 to neurons by exosomes was confirmed by examining effect of UD-P19 exosomes on differentiation of UD-P19 to neurons. Exposure to UD-P19 exosomes for 6 days had no impact on UD-P19 thus lending credence to P19N exosome-mediated neuronal differentiation of UD-P19. To our knowledge, this is the first report demonstrating exosome-mediated differentiation of pluripotent P19 cells into MAP2- and GluN2B-positive neurons.

The spatiotemporal expression of miRNAs is crucial during embryonic neurogenesis [47]. Some of these miRNAs/ncRNAs might be transferred through exosomes to stem cells prior to their neuronal differentiation. We therefore determined the identity of RNA packaged in UD-P19 and P19N exosomes. Small RNA-seq analysis identified a small set of 233 differentially expressed ncRNAs among several thousand ncRNAs common to UD-P19 and P19N exosomes. A Gene Ontology (GO) term analyses of differentially enriched RNAs in P19N exosomes using the MGI predicted associations of exosomal genes with terms related to neuron differentiation, regulation of synapse organization, long-term synaptic potentiation, basal dendrite, regulation of gene expression and alternative mRNA splicing via spliceosome, miRNA-mediated gene silencing, RISC complex, and several signaling pathways deemed essential for neuronal differentiation. The repertoire of exosomal ncRNAs identified in the present study could feed into the epigenetic regulatory networks involved in neuronal differentiation or proliferation of pluripotent cells [48]. These data and discussion below highlight the exceptional role of exosomes in cellular reprograming that is previously achieved through genetic modifications of cells e.g., by overexpressing transcription factors or microRNAs [49–51]. Exosomes thus present a novel alternative to genetic modifications-mediated cellular programming that is currently challenged by lack of sufficient knowledge of reprogramming factors, specificity, safety and efficacy of genetic modifications [52].

A literature search for several differentially enriched UD-P19 and P19N exosomal ncRNAs identified many notable ncRNAs previously implicated in neurogenesis or maintenance of stemness. For instance, miR-9 and let-7 were present only in P19N exosomes. MicroRNA-9 is important in neurogenesis, interneuron axon guidance and long-term synaptic potentiation [53–56]. Expression of let-7 is associated with neural differentiation and appears in E9.5 mouse embryos [57, 58]. Let-7 inhibits cell proliferation and cell cycle progression, and regulates terminal differentiation of neural stem cells [59, 60]. Malat1, Snord116, Rik201, miR-1839 levels were enriched in P19N exosomes. Malat1 is important in synaptogenesis and synapse function and regulates expression of synaptogenesis-related genes [61–63]. Snord116 is involved in the regulation of neuronal activity [64]. Rik201 regulates neural differentiation by modulating the expression of Sox6 and repressing the function of miR-96 [65]. MicroRNA-1839 is a Dicer-1-dependent non-canonical snoRNA and shares mRNA targets with let-7 [66]. It is thus clear that ncRNAs enriched in P19N exosomes are geared to drive differentiation of UD-P19 to P19N. Several ncRNAs such as GAS5, miR-6236, and polycistronic MIR17HG were especially enriched in UD-P19 exosomes. GAS5 regulates self-renewal and pluripotency of murine embryonic stem cells by enforcing a positive feedback network with pluripotency markers, Sox2, Oct4, Nanog, Tcl1, Esrrb, and Tet1 [67]. MIR17HG yields six miRs (miR-17, miR-18a, miR-19a, miR-20a, miR-19b-1, and miR-92a-1), has oncogenic properties and is involved in cell proliferation [68, 69]. Thus, enrichment of specific ncRNAs in UD-P19 exosomes can promote stem cell proliferation and maintenance of stem cell characteristics. Down-regulation of specific ncRNAs in P19N exosomes may have important implications in neurogenesis. We found P19N exosomes had reduced expression of miR-6236 and this reduction is known to augment neuronal development and neuro-regeneration [70]. These data highlight the importance of pro-neurogenic ncRNAs enrichment with a concomitant reduction/exclusion of pro-stemness ncRNAs in P19N exosomes for their role in inducing neurogenesis.

Implicating P19N exosomal ncRNAs in modulation of progenitor cell gene expression evokes the question whether exosomes have enough copies of neurogenic ncRNAs to be effective. There appears to be many potential answers to this question. One, many ncRNAs are effective in a dose-dependent manner. For instance, let-7 significantly down-regulates Dicer mRNA at low dose but targets c-myc mRNA at a higher dose [71]. Second, packaging ncRNAs in exosomes appears to be highly structured. P19N exosomes were packaged with co-targeting ncRNAs similar to brain tissue during neuronal differentiation [72]. Let-7 and miR-1839 that we found to be highly enriched in P19N exosomes, share mRNA targets and the target sites as the seed sequence of miR-1839 is shifted only by a single base [66]. Thus, multiple ncRNAs must synchronize their actions to effectively modulate gene expression for induction of neuronal differentiation. In addition, a safety net may be provided by exosomal proteins. Protein cargo of heterogeneous exosomes preparation from the hiPSC-derived neurons stimulate cell proliferation and neurogenesis through activation of signaling cascades [12]. Similar effects on neurogenesis are observed when homogeneous preparation of cyclin D1 enriched N2A neuronal exosomes are incubated with mouse embryonic stem cells (mESCs). Authors suggest that cyclin D1 is the crucial exosomal component driving the neuron differentiation of mESCs [13]. In both studies, the tissue culture medium contained B27 that contains retinol, a precursor of RA, thus B27 components might have primed cultured cells for induction of neurogenesis. Taken together, these data allude to concerted efforts of exosomal proteins and ncRNAs in neuronal differentiation irrespective of the model system used. Skipping a step during neuronal differentiation might lead to abnormal neurogenesis. For instance, the Wnt/STOP signaling is crucial in promoting neurogenesis in the mouse embryonic neocortex [73]. In P19 neurons, Wnt-1 expression coincides with RA-induction. UD-P19 do not express Wnt-1 but when overexpressed, Wnt-1 induces differentiation of UD-P19 to neurons albeit with aberrant neuronal processes [74]. A heterogeneous exosome preparation is thus likely to be more effective in modulating gene expression of pluripotent “stem” cells for their cellular differentiation as a recent review predicts that exosomes may carry either RNA-rich or protein-rich cargo due to their cellular site of origin [5].

One of the striking observations about ncRNAs was that many were present in their pre-miRNA state in UD-P19 and P19 exosomes. Similar enrichment of pre-miRNAs is reported in human breast milk exosomes [75]. When tested, milk exosomes miRNAs were resistant to harsh conditions including protection from ribonucleases [75, 76]. Packaging P19N exosomes with pre-ncRNAs may be an advantage to protect them from degradation before reaching recipient cells as mature miRNAs are short-lived with half-lives ranging from few minutes to hours in brain/neurons [77]. However, in most other tissues, mature miRNAs are quite stable with half-lives ranging from several hours to days [78]. An alternative scenario could suggest that exosomal pre-miRNAs may be functionally important during development. For instance, ectopic expression of pre-miR-181a-1 in thymic progenitor cells induces their differentiation into CD4 and CD8 double-positive T cells [79]. The RNase III enzyme, Dicer-1 cleaves pre-miRNAs to form mature miRNAs in the cytoplasm [80]. Functionally active Dicer-1 is present in exosomes. Incubation of breast cancer cell exosomes at 37 °C leads to cleavage of pre-miRNAs to their mature form [32]. Dicer-1 was abundantly present in P19N exosomes. Although we did not examine processing of pre-miRNAs to form mature miRNAs, it is likely that pre-ncRNAs in P19N exosomes undergo maturation prior to/after entering the recipient cells, UD-P19. Once internalized, mature ncRNAs can hitch-hike cellular argonaute-2 protein to form RNA-induced silencing complex (RISC) to modulate gene expression of recipient cells [81]. Incidentally, argonaute-2 protein was absent in P19N exosomes. Understanding contributions of exosomal ncRNAs is crucial to decipher the sequence of events that either support cells maintain their stem cell status or exit proliferation and proceed towards differentiation. Currently, experiments employing various strategies (e.g., CRISPR-Cas9 genome editing technology) are in progress in our laboratory to decode and understand the precise functional role of P19N exosomal ncRNAs in modulating gene expression to drive the differentiation of UD-P19 cells to neurons.

In conclusion, UD-P19 and P19N released exosomes with normal characteristics and had overlapping but distinct ncRNA cargos. Besides common ncRNAs, exosomes from each cell were enriched with specific ncRNAs that were either pro-stemness (proliferation and maintenance of stem cell characteristics) or pro-neurogenic (induces neuronal differentiation). Internalization of P19N exosomes induced differentiation of UD-P19 to MAP2- and GluN2B-positive neurons. Differentiation of neurons using exosomes has immense therapeutic applications from spinal cord and brain injury to neurodegenerative diseases.

## Supplementary Information

Supplementary Figure S1 An overview to UD-P19 and P19N culture conditions, and retinoic acid mediated differentiation of UD-P19 to P19 neurons. Cell culture methods used in this study have been established and published previously by our laboratory. **a**) A brief overview to steps involved in culturing 60-70% confluent UD-P19 in serum free medium. Cells were passaged at 1:5 ratio yielding ~50% confluent UD-P19 that were cultured in α-MEM containing 10% FBS. In 24h, cells became 60-70% confluent. They were washed and fed α-MEM containing N2 supplement (serum free medium). After 24h, conditioned medium (CM) was collected and processed immediately for isolation of exosomes. **b**) UD-P19 from one 75 cm^2^ flask were trypsinized and plated in a 10 mm petri plate in α-MEM containing 5% FBS and 1μM retinoic acid (RA). Within 2d, cells began to aggregate and formed free floating embryoid bodies (EBs). EBs were collected by low speed centrifugation and plated in a new petri plate. They were cultured for 2d in α-MEM containing 5% FBS and 1μM RA. At the end of RA treatment, EBs were washed, triturated gently and plated in poly-L-lysine coated 75 cm^2^ flask pre-incubated with laminin for 1h. EBs were cultured for 4d in neurobasal medium (NB) containing N2 supplement and glutamine with change of medium after 2d. After 4d, cells were cultured in NB/B27/N2/glutamine medium. Two days later, cells were fed fresh NB/B27/N2/glutamine medium. At the end of 2d, CM was recovered and processed immediately for isolation of exosomes. Culture medium for all cells contained 1x antibiotic/anti-mycotic solution (MilliporeSigma). (PNG 220 kb)

High resolution image (TIF 141 kb)

Supplementary Figure S2 Flow chart showing centrifugation steps with rotor and k-factor for isolation of UD-P19 and P19N exosomes by differential ultracentrifugation. (PNG 97 kb)

High resolution image (TIF 56 kb)

Supplementary Figure S3 Solubilization of exosomes for Western blotting. Not all lysis buffers were compatible with detection of exosomal marker proteins. Lysis buffer, solubilization of exosomes, and storage conditions of solubilized samples for Western blotting were optimized. **a:** Exosomes volume containing 50 μg protein were diluted to 2 mL with PBS ultracentrifuged to pellet down the exosomes. Exosome pellet was suspended directly in 1x RIPA buffer containing anti-proteases to prevent labile CD63 protein degradation. Exosomes suspended in RIPA buffer were incubated at 37°C to solubilize exosome membranes. Samples were gently mixed every 5 min. At the end of incubation, samples were centrifuged, mixed with appropriate volume of 5X Laemmli’s sample buffer and heat-denatured for 3 min in boiling water. Solubilized exosomes were stored at -70°C if not processed immediately for Western blotting. **b:** Exosomes in PBS containing a known amount of protein were mixed with 5X Laemmli’s sample buffer and heat-denatured for 3 min in boiling water. Solubilized exosomes were stored at -20°C if not processed immediately for Western blotting. Protein amount required for every exosomal marker protein was optimized after selection of optimal lysis buffer. (PNG 266 kb)

High resolution image (TIF 167 kb)

Supplementary Figure S4 Appearance of exosome pellet after ultracentrifugation. Exosomes appeared as a translucent pellet (dotted circle) in centrifuge tube after quick removal of supernatant following ultracentrifugation and observing the tube against light. (PNG 693 kb)

High resolution image (TIF 707 kb)

Supplementary Figure S5 Optimization of lysis buffer and primary antibodies to CD9, CD81, tsg101, and cytochrome C for detection of the respective cognate protein by Western blotting. Fetal cortical lysate (FCN) proteins were separated on 8 or 10% polyacrylamide gels, blotted, and probed with one of the primary antibodies (CD9, CD81, tsg101 or cytochrome C). Immunoreactive proteins (indicated by black arrows) were detected using ECL plus reagent and scanning on a PhosphorImager. CD9 and CD81 immunoblots: Lanes 1 and 2: lysate proteins dissolved in RIPA buffer; Lanes 3 and 4: lysate proteins dissolved in Laemmli’s buffer; Lanes 1 and 3: 20 μg protein; Lanes 2 and 4: 30 μg protein; tsg101 and cytochrome C immunoblots: Lanes1 to 5: Increasing concentration of lysate protein (5, 10, 15, 20, and 25 μg respectively) was loaded on the gel. Pre-stained protein molecular weight markers (NEB) were included in all gels (Lane M) and their size is indicated in kDa on the left. (PNG 742 kb)

High resolution image (TIF 724 kb)

Supplementary Figure S6 Exosomes were processed for immuno-EM by indirect method using anti-CD63 and gold (18 nm) conjugated secondary antibody. Representative electron micrograph of exosomes incubated with 1% donkey serum in lieu of anti-CD63 (negative control) (Top image). Representative electron micrograph of exosomes incubated with anti-CD63 showed surface localization of CD63 protein on UD-P19 exosomes (bottom image). Electron micrograph of region highlighted by red square was captured at higher magnification to show gold particles bound to exosome (inset in the bottom electron micrograph). Inset image is shown in Figure 2e with magnification. Scale bar = 200 nm. (PNG 4926 kb)

High resolution image (TIF 6702 kb)

Supplementary Figure S7 P19N exosomes are internalized by UD-P19 and P19N. Cells cultured with Dil-labeled P19N exosomes (red) for 24h were fixed, counterstained with nuclear stain, DAPI (blue), and examined under confocal microscope (Zeiss LSM-700). Cells were optically sliced (z-stacked) using an EC Plan-Neofluar 40x/1.30 NA objective to determine intracellular localization of Dil-labeled exosomes. **a**: Representative z-stacks of UD-P19 incubated with Dil-labeled exosomes (11 μm focal distance; 1 μm/step) (images 1-12). Cytosolic Dil fluorescence was maximum in optical sections between 4 and 7 μm. The z-stacks were processed into maximum intensity projections for DAPI (blue), Dil (red), and both channels merged together (Merge). Scale bar = 20 μm. **b**: Representative z-stacks of P19N incubated with Dil-labeled P19N exosomes (6 μm focal distance; 0.5 μm/step) (images 1-12). Cytosolic Dil fluorescence was maximum in optical sections between 2.5 and 3.5 μm. The z-stacks were processed into maximum intensity projections for bright field (grey color), DAPI (blue), Dil (red), and DAPI/red merged fluorescence (Merge). Scale bar = 20 μm. **c and d**: As controls, UD-P19 (c) and P19N (d) were independently incubated for 24h with Dil/DPBS mix processed in the same manner as Dil/exosomes. No Dil fluorescence was observed in control cultures. Scale bar = 50 μm. (DOCX 2665 kb)

Supplementary Figure S8 Exosome-mediated differentiation of UD-P19 to neurons. Representative tiled images of undifferentiated P19 cells cultured in the absence (Control – No Exo) or presence (Exo Treated) of P19N exosomes for 2 days (**a**), 4 days (**b**), and 6 days (**c**) were captured with a Zeiss confocal microscope using 10X (Plan-Neofluar 10x/0.30) objective. All images were stitched using Stitch feature of Zen software. Cultures terminated at each time point were processed for immunostaining of neurites with MAP2 (green) and nuclei with DAPI (blue). Formation of embryoid bodies following P19N exosome exposure is indicated by red arrowheads and MAP-2 positive cells by white arrows. Scale bar = 500 μm. (DOCX 1564 kb)

Supplementary Figure S9 Exosomal RNA analysis on BioAnalyzer. Representative electropherograms were obtained by electrophoresing denatured exosomal RNA in Agilent’s small RNA chip on BioAnalyzer. The 4nt RNA marker was included in every well. Low molecular weight RNA ladder was included in the run. Arbitrary fluorescence units (FU) were plotted as a function of RNA size in nucleotides (nt). **a**: UD-P19 exosome RNA, **b**: P19N exosome RNA. (PNG 169 kb)

High resolution image (TIF 329 kb)

Supplementary Figure S10 Quality control analysis of barcoded cDNA libraries. Electropherograms of six double-stranded barcoded cDNA libraries (UD-P19 1-3 = three biological replicates of UD-P19 exosomes and P19N 1-3 = three biological replicates of P19N exosomes) obtained on BioAnalyzer using a High Sensitivity DNA chip. Biological replicate for each cell population is indicated on the left. Arbitrary fluorescence units (FU) were plotted as a function of DNA size in base pairs (bp). (PNG 295 kb)

High resolution image (TIF 340 kb)

Supplementary Figure S11 Flow chart showing filtration of differentially enriched/depleted ncRNAs in exosomes. (PNG 69 kb)

High resolution image (TIF 91 kb)

Supplementary Figure S12 Two-way hierarchical clustering of salient transcripts. The heat map was generated using the log_2_ transformed expression values of the differentially enriched transcripts purified from UD-P19 and P19N exosomes. To reduce complexity, only 218 transcripts with mean expression values ≥ 10 in UD-P19 or P19N were included in the heat map. The columns represent three biological replicates per parent cell. Each row of circles represents a single transcript. Transcript enrichment is indicated in pink and depletion in blue. The values in the heat map are mapped to colors using the minimum and maximum of each row independently. The numbers in the circles represent the log_2_ transformed expression values. (PDF 1108 kb)

Supplementary Table TS1 (DOCX 12 kb)

Supplementary Table TS2 Optimization of sample preparation and Western blot conditions. This table provides details of Western blot conditions optimized and used to detect various proteins in exosomes and cell lysates. Notes to superscripted numbers are provided below the table. (DOCX 14 kb)

Supplementary Table TS3 (XLSB 9325 kb)

Supplementary Table TS4 (XLSB 8254 kb)

Supplementary Table TS5 List of differentially enriched/depleted ncRNAs in UD-P19 and P19N exosomes. Non-coding RNAs identified in UD-P19 and P19N exosomes were filtered in a step-wise manner to obtain number and list of differentially enriched/depleted ncRNAs in UD-P19 and P19N exosomes. Three filters applied for data analysis are shown in Supplementary Figure S10. (XLS 119 kb)

Supplementary Table TS6 (XLSX 29 kb)

## Data Availability

All original data is available upon request from the communicating author, Meena Kumari.

## Code availability

Software (ImageJ) used for image analysis is available free to everyone on a public platform. Statistical analysis of data was performed using GraphPad that is available from the company for a fee. No other software or code was developed for the study presented in this manuscript.

## Declarations

### Data Sharing

The small RNA-Seq data discussed in this publication has been deposited in NCBI's Gene Expression Omnibus [82, 83] and are accessible through GEO Series accession number GSE152655 (https://www.ncbi.nlm.nih.gov/geo/query/acc.cgi?acc=GSE152655).

### Health and Safety

Authors confirm that all mandatory health and safety procedures have been complied within the course of conducting experimental work reported in our manuscript. Preparation and use of 4% formaldehyde, Trump’s fixative, and uranyl acetate was carried out in the fume hood. All radioactive work was carried out in the designated space within our laboratories. All biohazard chemicals were disposed through the environmental safety office of Kansas State University.
