# Supplementary figures and images for: Exosomes induce neurogenesis of pluripotent P19 cells
Source: Stem Cell Rev Rep. 2023 Feb 22;19(5):1152–76. doi: 10.1007/s12015-023-10512-6 (PMC10366297; doi:10.1007/s12015-023-10512-6)

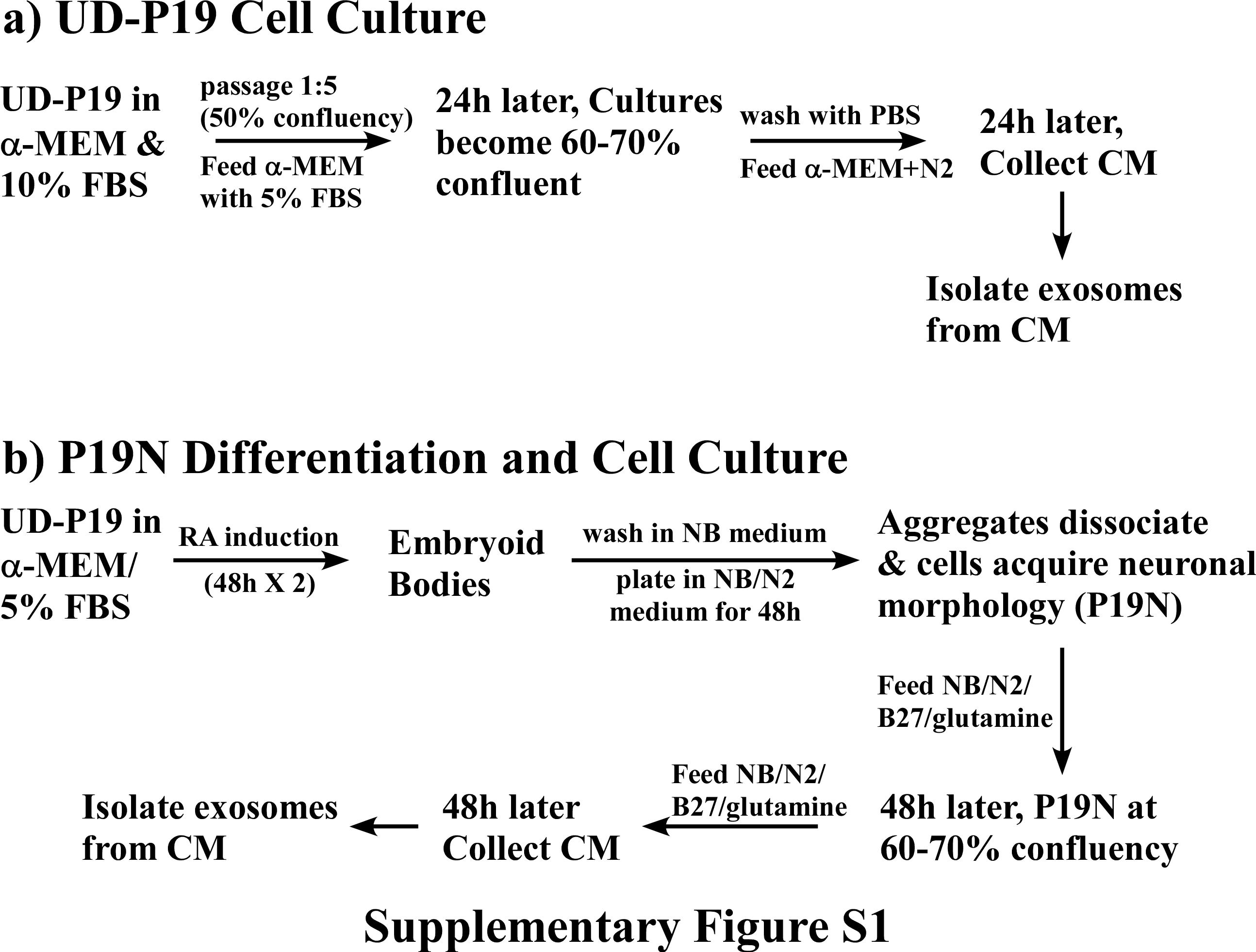

Supplement: Supplementary file 1 — An overview to UD-P19 and P19N culture conditions, and retinoic acid mediated differentiation of UD-P19 to P19 neurons. Cell culture methods used in this study have been established and published previously by our laboratory. a) A brief overview to steps involved in culturing 60-70% confluent UD-P19 in serum free medium. Cells were passaged at 1:5 ratio yielding ~50% confluent UD-P19 that were cultured in α-MEM containing 10% FBS. In 24h, cells became 60-70% confluent. They were washed and fed α-MEM containing N2 supplement (serum free medium). After 24h, conditioned medium (CM) was collected and processed immediately for isolation of exosomes. b) UD-P19 from one 75 cm2 flask were trypsinized and plated in a 10 mm petri plate in α-MEM containing 5% FBS and 1μM retinoic acid (RA). Within 2d, cells began to aggregate and formed free floating embryoid bodies (EBs). EBs were collected by low speed centrifugation and plated in a new petri plate. They were cultured for 2d in α-MEM containing 5% FBS and 1μM RA. At the end of RA treatment, EBs were washed, triturated gently and plated in poly-L-lysine coated 75 cm2 flask pre-incubated with laminin for 1h. EBs were cultured for 4d in neurobasal medium (NB) containing N2 supplement and glutamine with change of medium after 2d. After 4d, cells were cultured in NB/B27/N2/glutamine medium. Two days later, cells were fed fresh NB/B27/N2/glutamine medium. At the end of 2d, CM was recovered and processed immediately for isolation of exosomes. Culture medium for all cells contained 1x antibiotic/anti-mycotic solution (MilliporeSigma). (PNG 220 kb) [file 12015_2023_10512_Fig7_ESM.png]

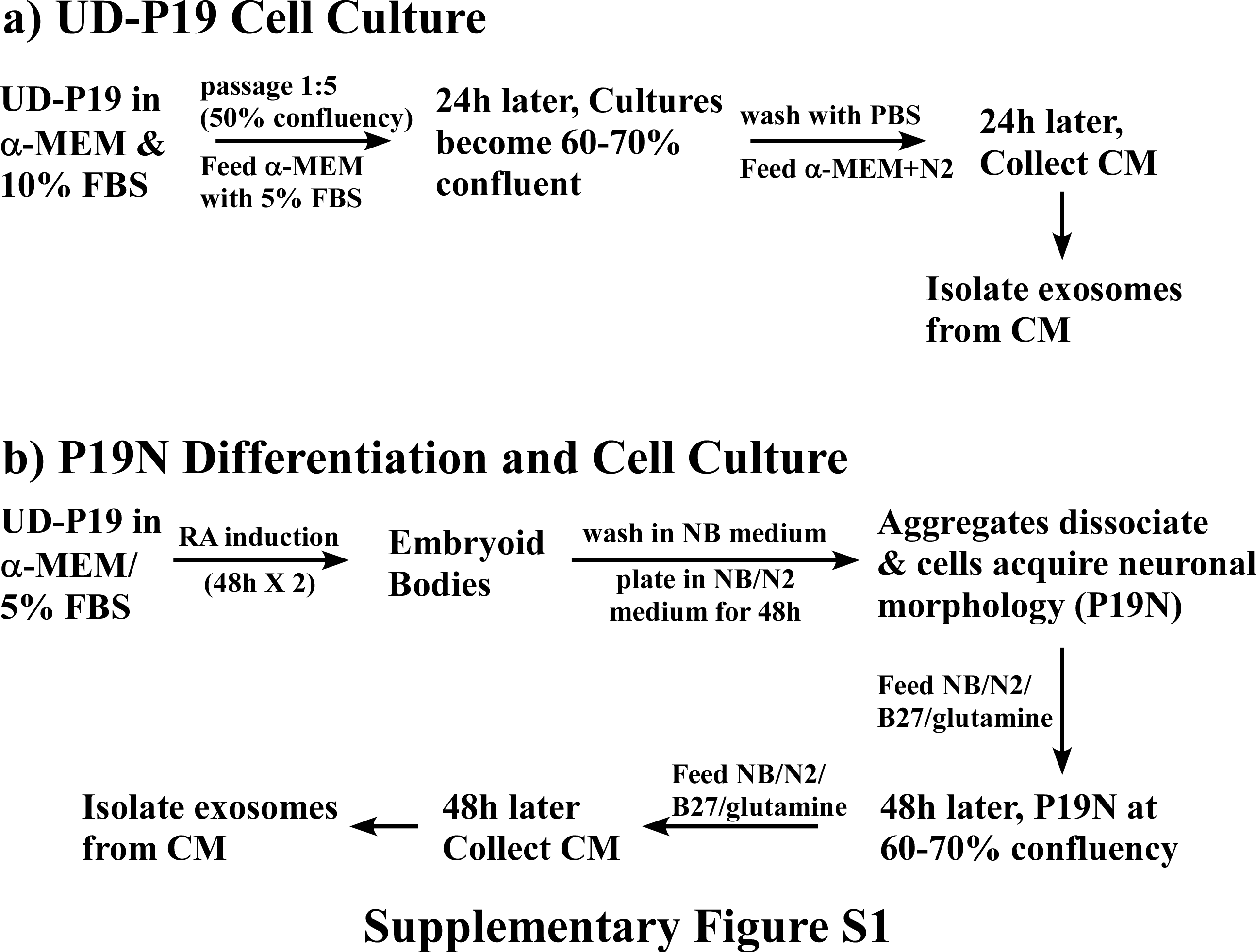

Supplement: Supplementary file 2 — High resolution image (TIF 141 kb) [file 12015_2023_10512_MOESM1_ESM.tif]

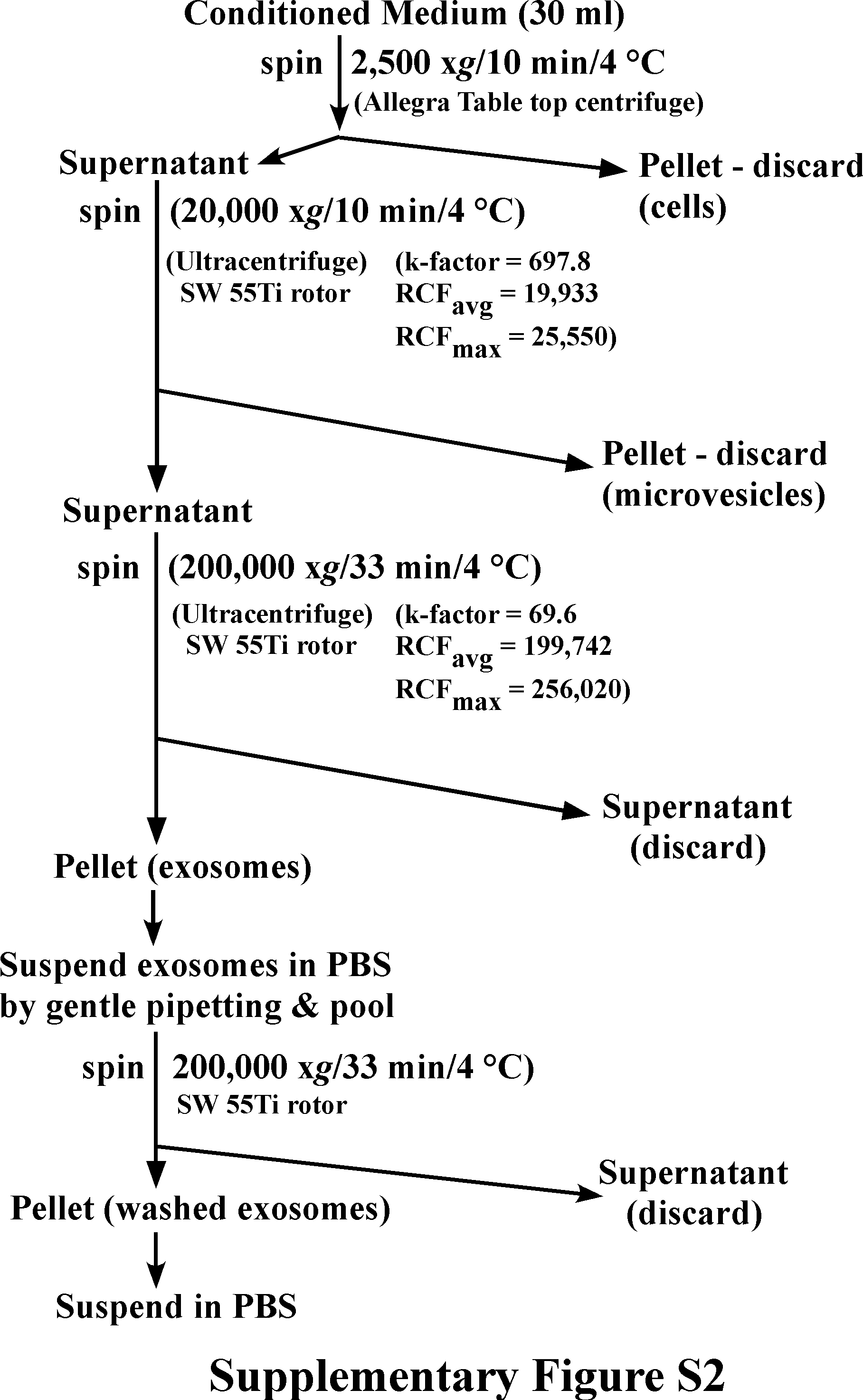

Supplement: Supplementary file 3 — Flow chart showing centrifugation steps with rotor and k-factor for isolation of UD-P19 and P19N exosomes by differential ultracentrifugation. (PNG 97 kb) [file 12015_2023_10512_Fig8_ESM.png]

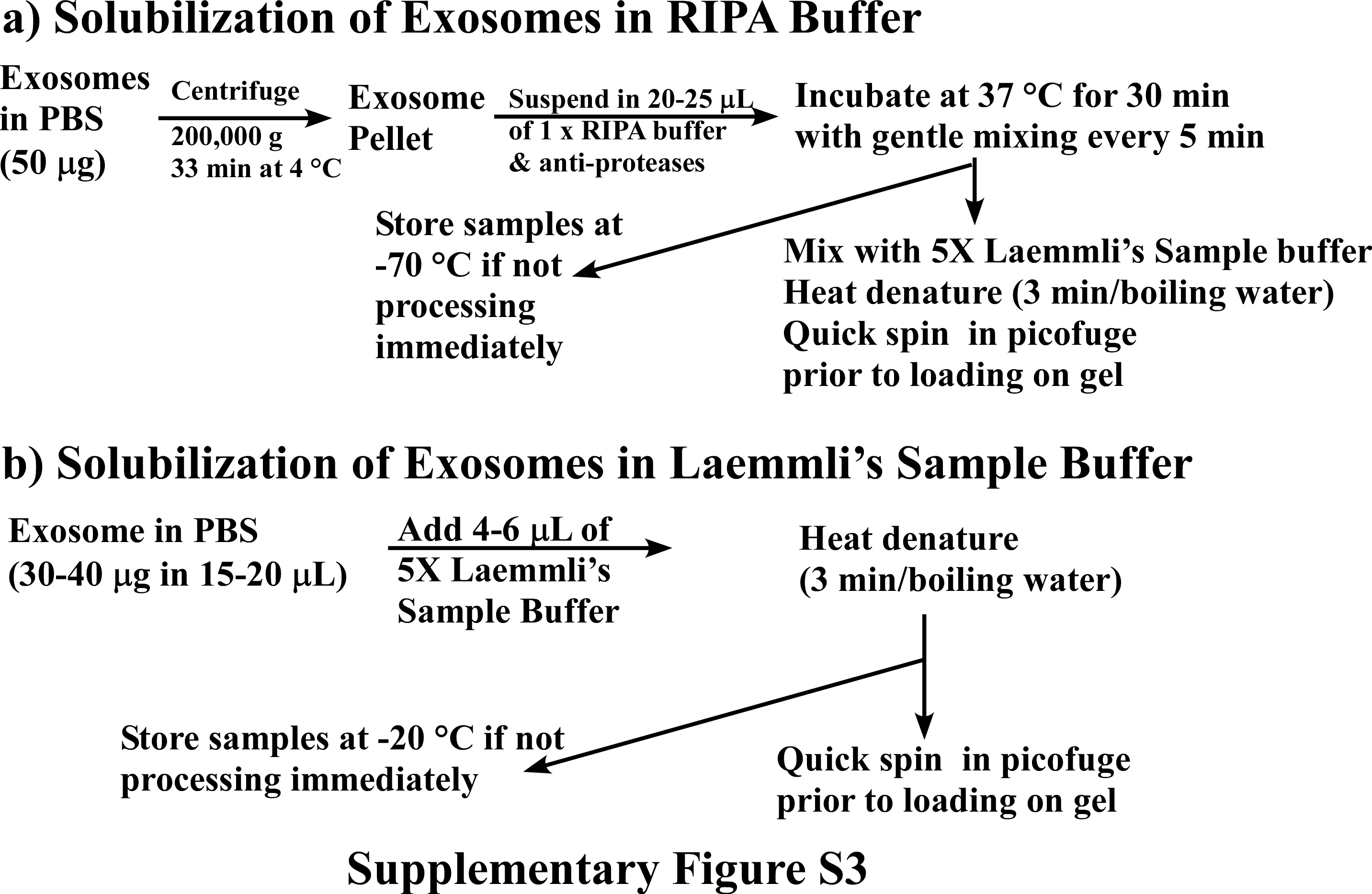

Supplement: Supplementary file 5 — Solubilization of exosomes for Western blotting. Not all lysis buffers were compatible with detection of exosomal marker proteins. Lysis buffer, solubilization of exosomes, and storage conditions of solubilized samples for Western blotting were optimized. a: Exosomes volume containing 50 μg protein were diluted to 2 mL with PBS ultracentrifuged to pellet down the exosomes. Exosome pellet was suspended directly in 1x RIPA buffer containing anti-proteases to prevent labile CD63 protein degradation. Exosomes suspended in RIPA buffer were incubated at 37°C to solubilize exosome membranes. Samples were gently mixed every 5 min. At the end of incubation, samples were centrifuged, mixed with appropriate volume of 5X Laemmli’s sample buffer and heat-denatured for 3 min in boiling water. Solubilized exosomes were stored at -70°C if not processed immediately for Western blotting. b: Exosomes in PBS containing a known amount of protein were mixed with 5X Laemmli’s sample buffer and heat-denatured for 3 min in boiling water. Solubilized exosomes were stored at -20°C if not processed immediately for Western blotting. Protein amount required for every exosomal marker protein was optimized after selection of optimal lysis buffer. (PNG 266 kb) [file 12015_2023_10512_Fig9_ESM.png]

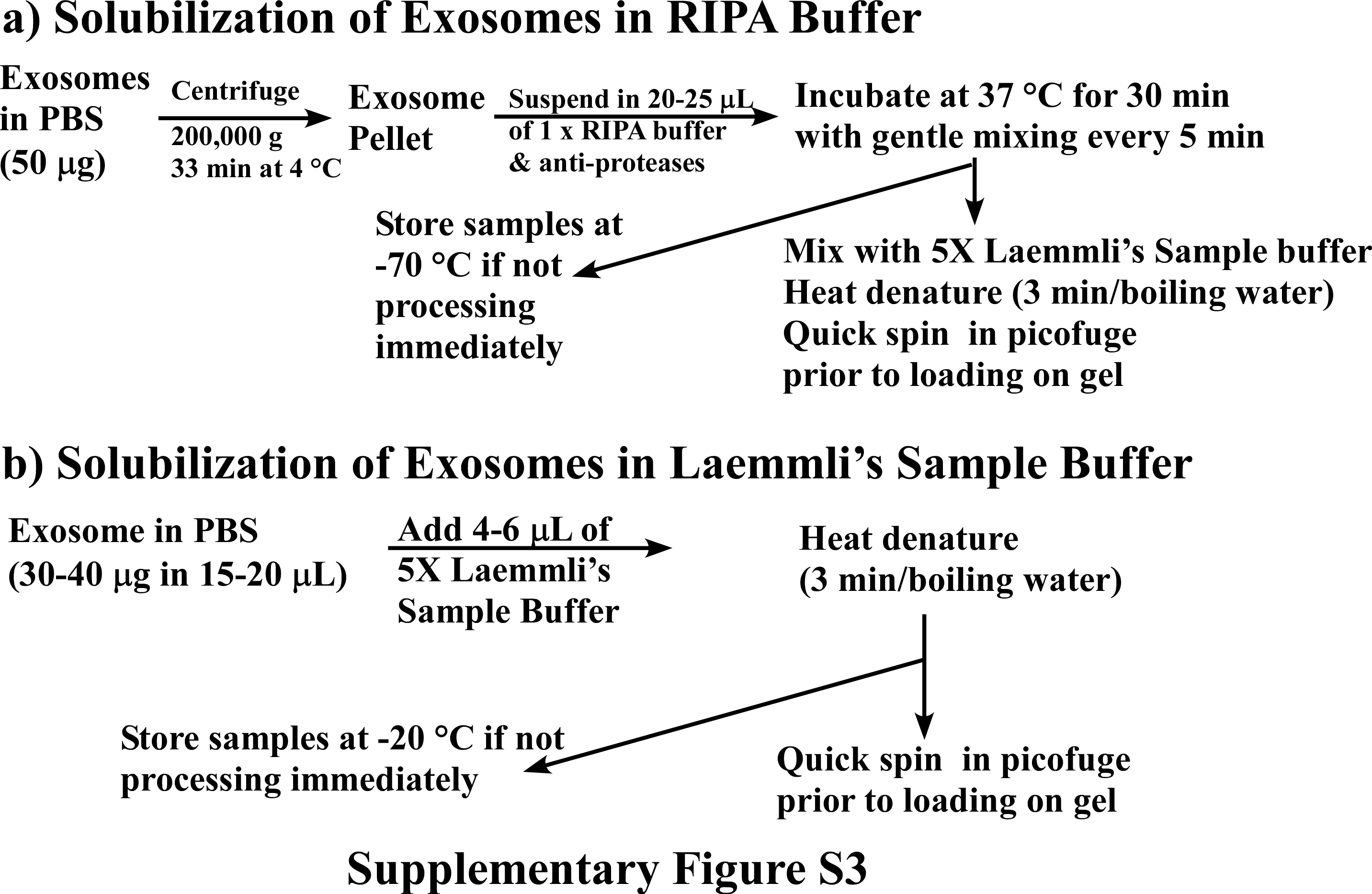

Supplement: Supplementary file 6 — High resolution image (TIF 167 kb) [file 12015_2023_10512_MOESM3_ESM.tif]

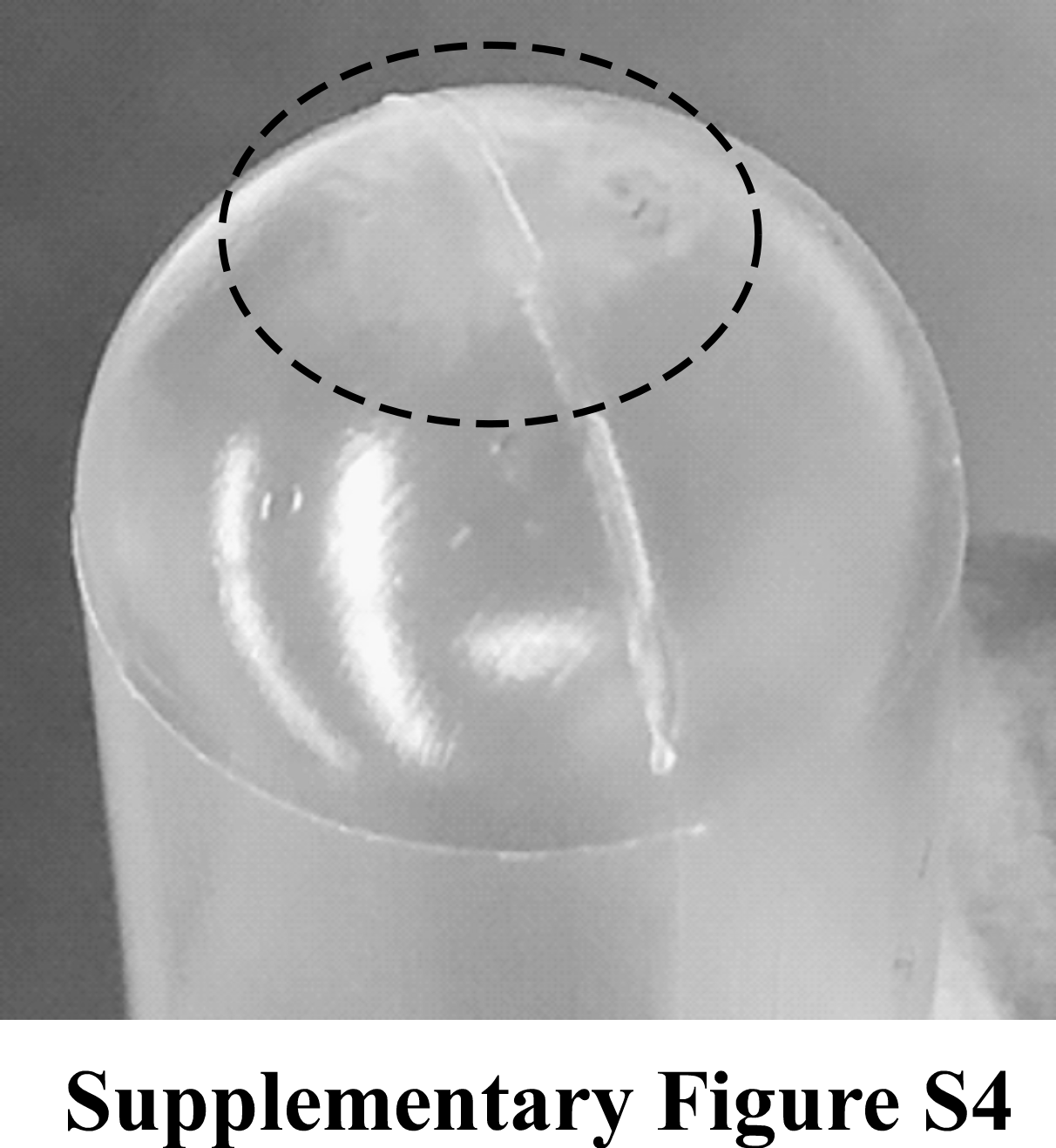

Supplement: Supplementary file 7 — Appearance of exosome pellet after ultracentrifugation. Exosomes appeared as a translucent pellet (dotted circle) in centrifuge tube after quick removal of supernatant following ultracentrifugation and observing the tube against light. (PNG 693 kb) [file 12015_2023_10512_Fig10_ESM.png]

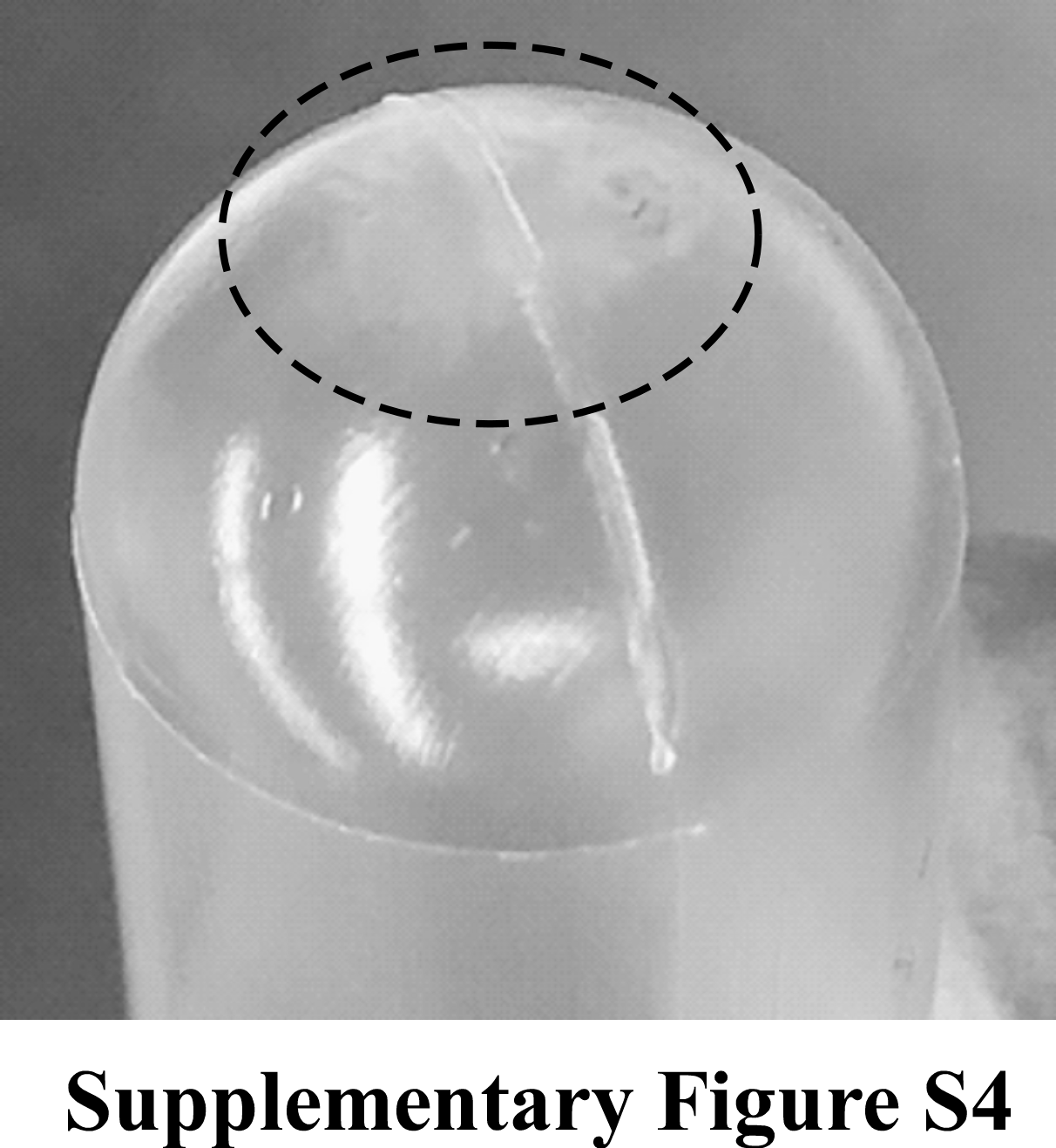

Supplement: Supplementary file 8 — High resolution image (TIF 707 kb) [file 12015_2023_10512_MOESM4_ESM.tif]

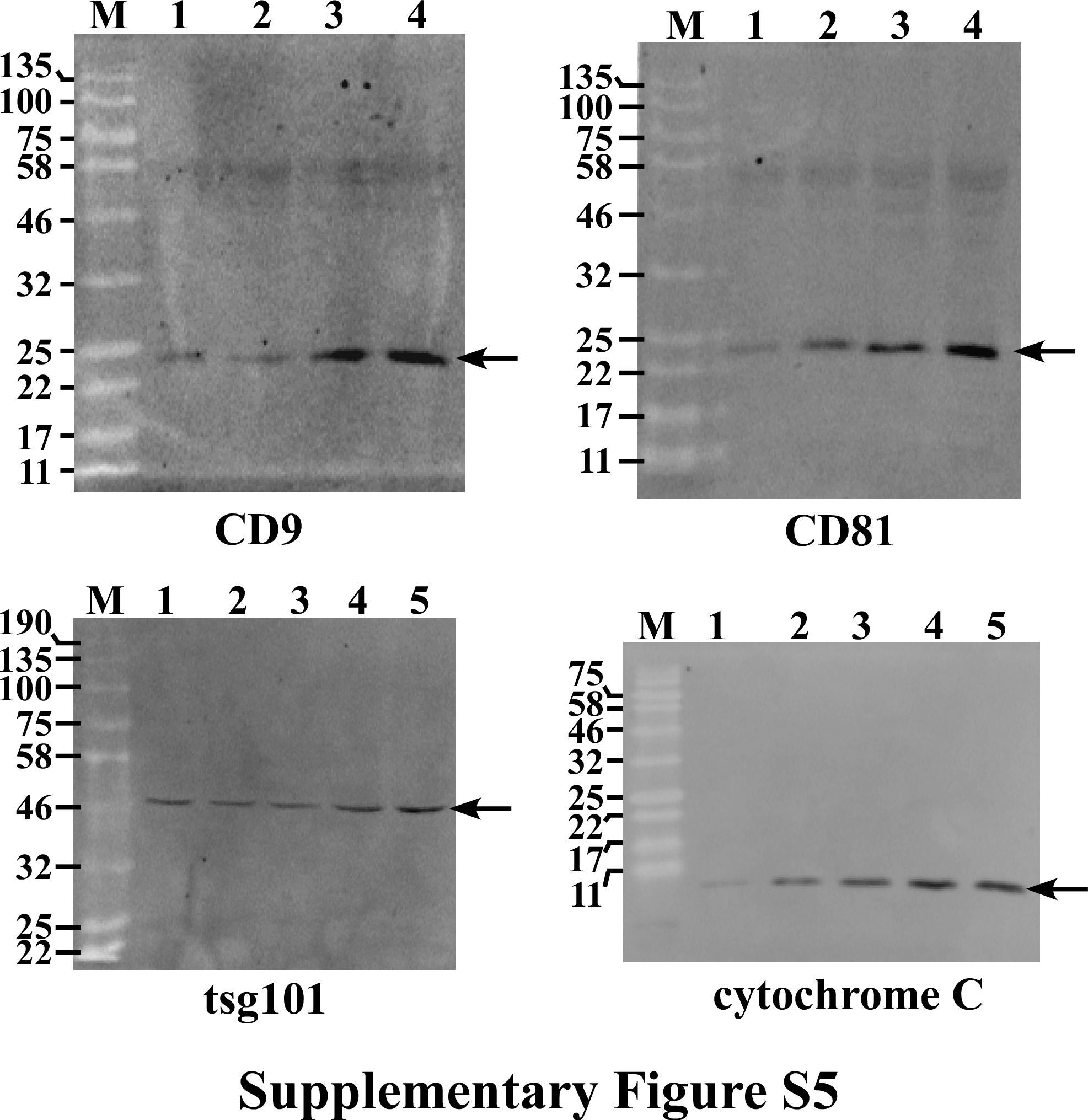

Supplement: Supplementary file 9 — Optimization of lysis buffer and primary antibodies to CD9, CD81, tsg101, and cytochrome C for detection of the respective cognate protein by Western blotting. Fetal cortical lysate (FCN) proteins were separated on 8 or 10% polyacrylamide gels, blotted, and probed with one of the primary antibodies (CD9, CD81, tsg101 or cytochrome C). Immunoreactive proteins (indicated by black arrows) were detected using ECL plus reagent and scanning on a PhosphorImager. CD9 and CD81 immunoblots: Lanes 1 and 2: lysate proteins dissolved in RIPA buffer; Lanes 3 and 4: lysate proteins dissolved in Laemmli’s buffer; Lanes 1 and 3: 20 μg protein; Lanes 2 and 4: 30 μg protein; tsg101 and cytochrome C immunoblots: Lanes1 to 5: Increasing concentration of lysate protein (5, 10, 15, 20, and 25 μg respectively) was loaded on the gel. Pre-stained protein molecular weight markers (NEB) were included in all gels (Lane M) and their size is indicated in kDa on the left. (PNG 742 kb) [file 12015_2023_10512_Fig11_ESM.png]

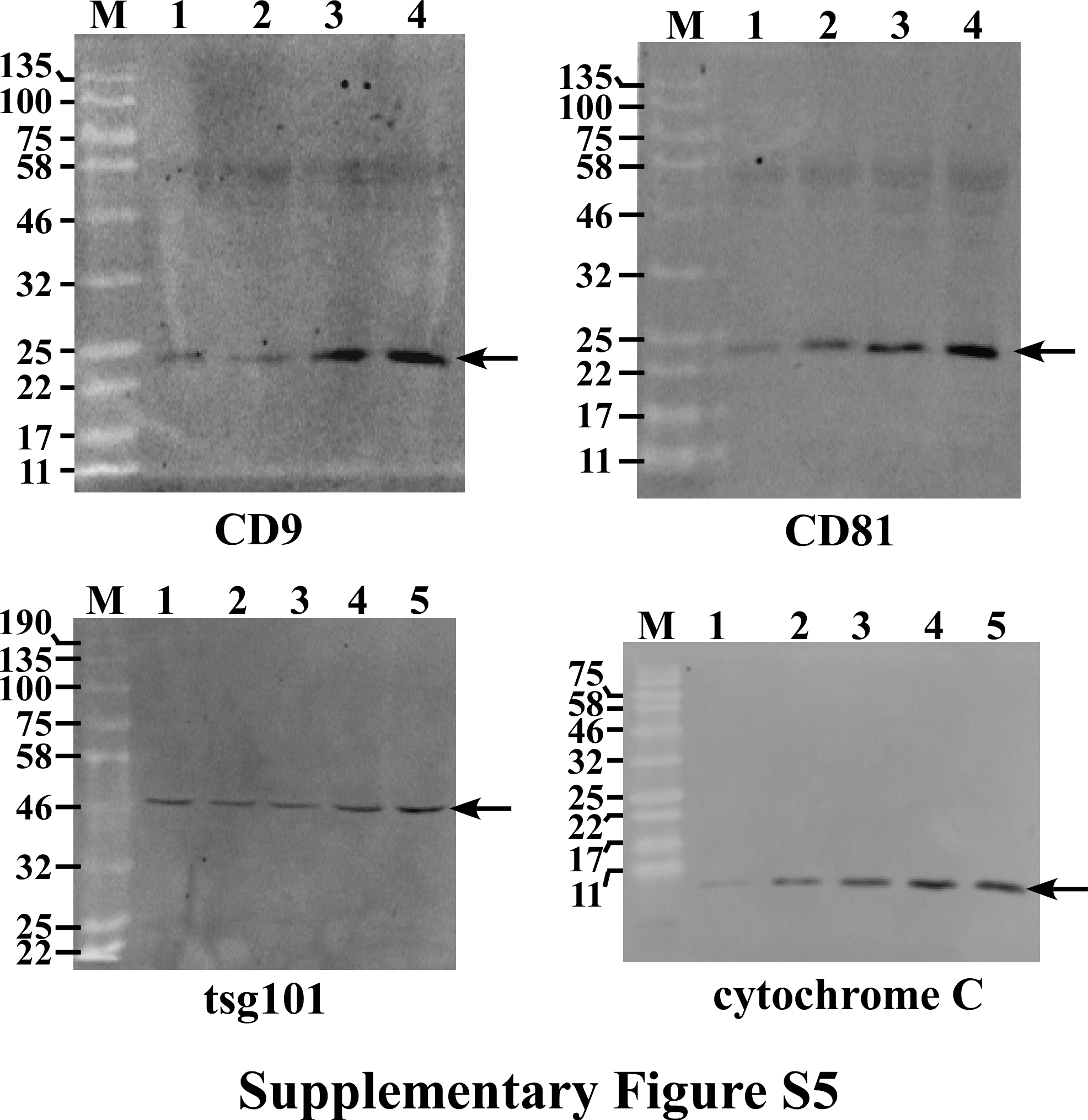

Supplement: Supplementary file 10 — High resolution image (TIF 724 kb) [file 12015_2023_10512_MOESM5_ESM.tif]

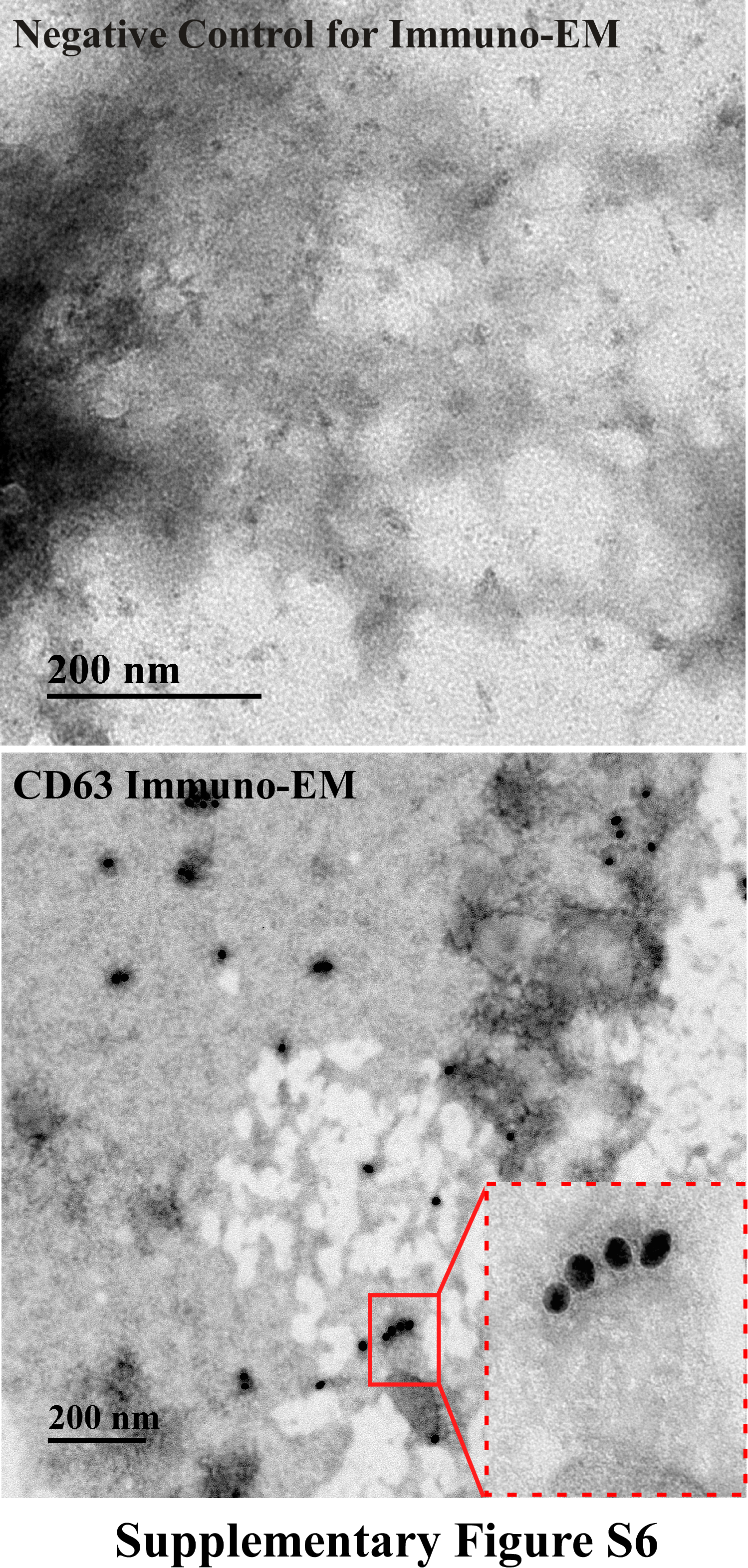

Supplement: Supplementary file 11 — Exosomes were processed for immuno-EM by indirect method using anti-CD63 and gold (18 nm) conjugated secondary antibody. Representative electron micrograph of exosomes incubated with 1% donkey serum in lieu of anti-CD63 (negative control) (Top image). Representative electron micrograph of exosomes incubated with anti-CD63 showed surface localization of CD63 protein on UD-P19 exosomes (bottom image). Electron micrograph of region highlighted by red square was captured at higher magnification to show gold particles bound to exosome (inset in the bottom electron micrograph). Inset image is shown in Figure 2e with magnification. Scale bar = 200 nm. (PNG 4926 kb) [file 12015_2023_10512_Fig12_ESM.png]

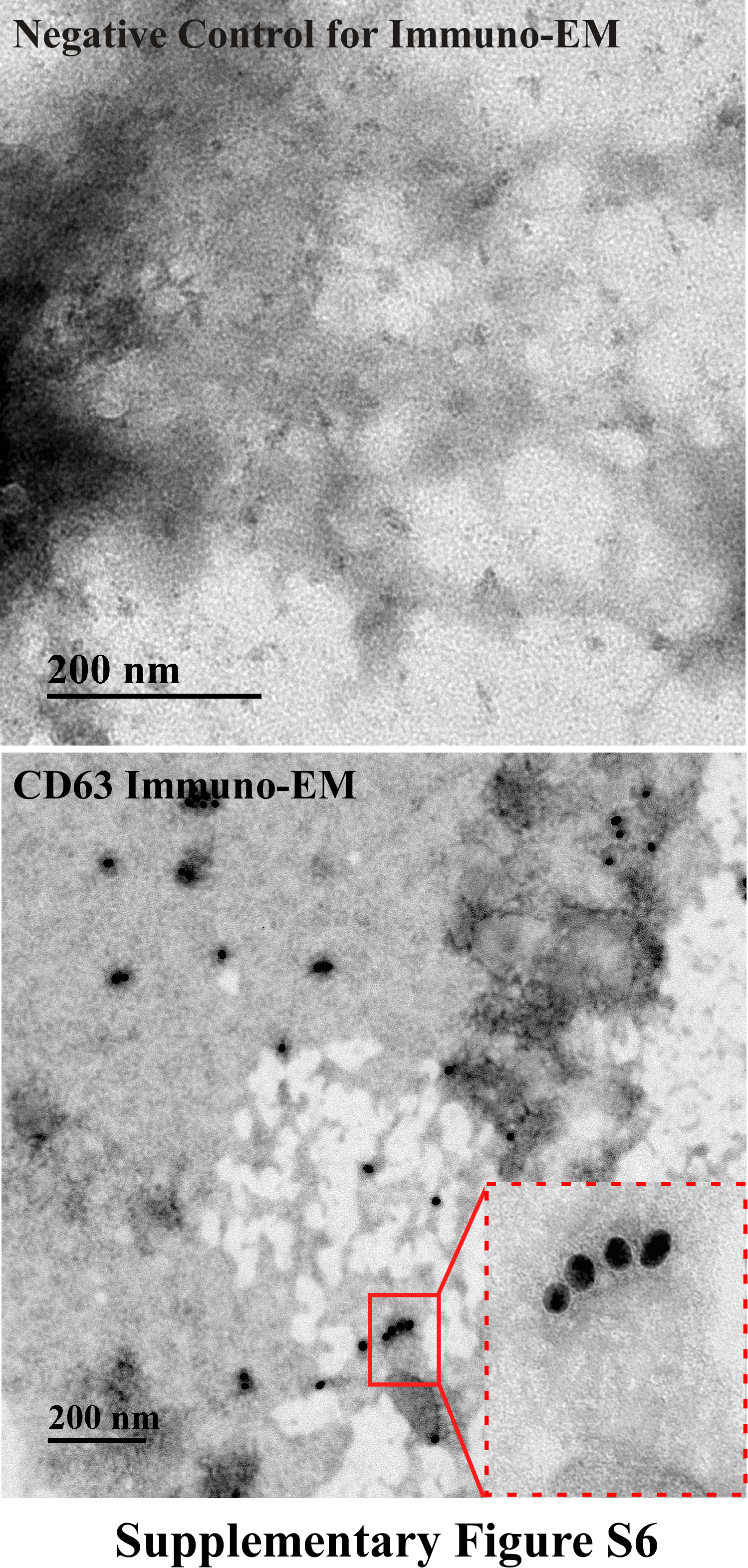

Supplement: Supplementary file 12 — High resolution image (TIF 6702 kb) [file 12015_2023_10512_MOESM6_ESM.tif]

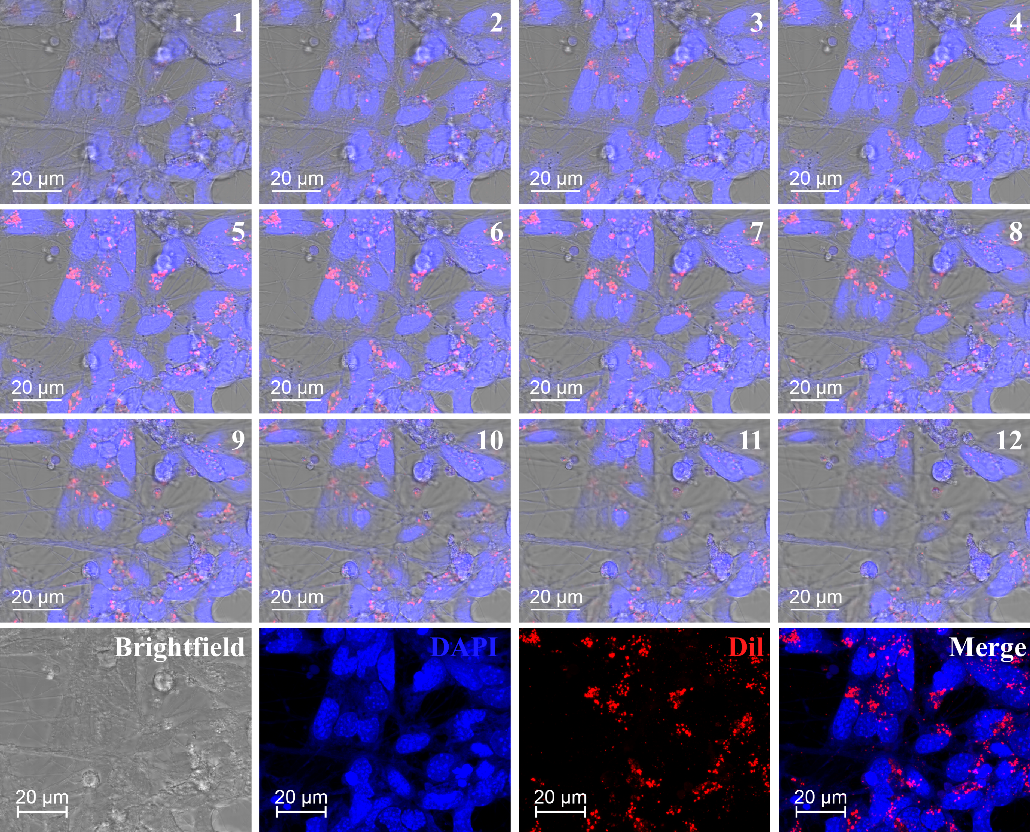
**
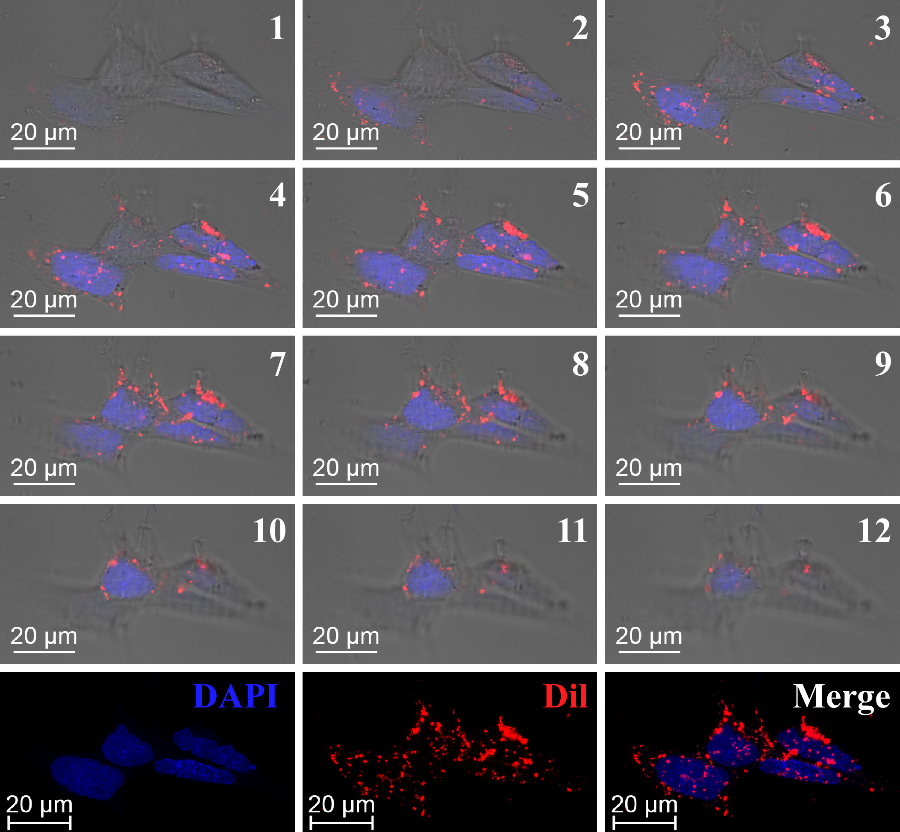
** **S7a S7b**


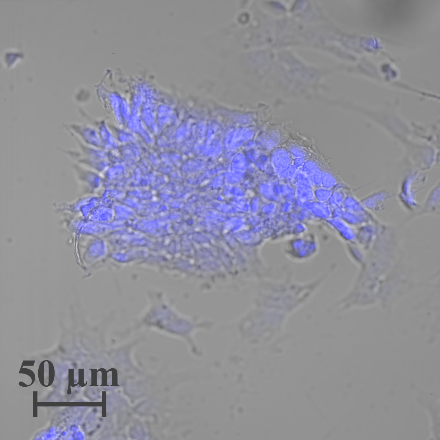

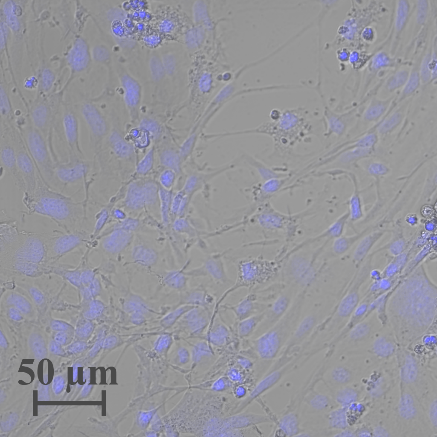


**S7c S7d**

**Supplementary Figure S7**

Supplement: Supplementary file 13 — P19N exosomes are internalized by UD-P19 and P19N. Cells cultured with Dil-labeled P19N exosomes (red) for 24h were fixed, counterstained with nuclear stain, DAPI (blue), and examined under confocal microscope (Zeiss LSM-700). Cells were optically sliced (z-stacked) using an EC Plan-Neofluar 40x/1.30 NA objective to determine intracellular localization of Dil-labeled exosomes. a: Representative z-stacks of UD-P19 incubated with Dil-labeled exosomes (11 μm focal distance; 1 μm/step) (images 1-12). Cytosolic Dil fluorescence was maximum in optical sections between 4 and 7 μm. The z-stacks were processed into maximum intensity projections for DAPI (blue), Dil (red), and both channels merged together (Merge). Scale bar = 20 μm. b: Representative z-stacks of P19N incubated with Dil-labeled P19N exosomes (6 μm focal distance; 0.5 μm/step) (images 1-12). Cytosolic Dil fluorescence was maximum in optical sections between 2.5 and 3.5 μm. The z-stacks were processed into maximum intensity projections for bright field (grey color), DAPI (blue), Dil (red), and DAPI/red merged fluorescence (Merge). Scale bar = 20 μm. c and d: As controls, UD-P19 (c) and P19N (d) were independently incubated for 24h with Dil/DPBS mix processed in the same manner as Dil/exosomes. No Dil fluorescence was observed in control cultures. Scale bar = 50 μm. (DOCX 2665 kb) [file 12015_2023_10512_MOESM7_ESM.docx]

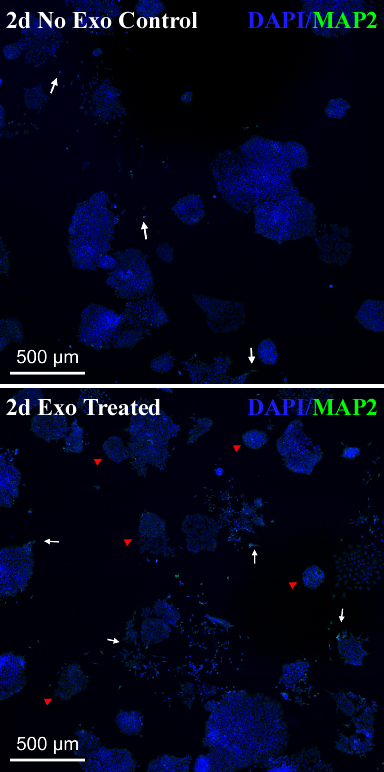


**Supplementary Figure S8a**


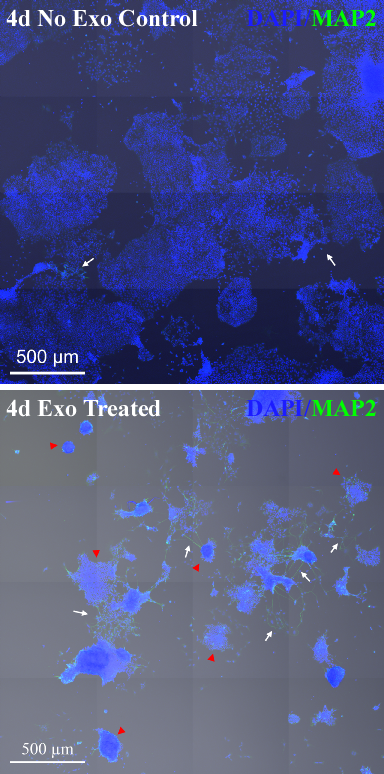


**Supplementary Figure S8b**


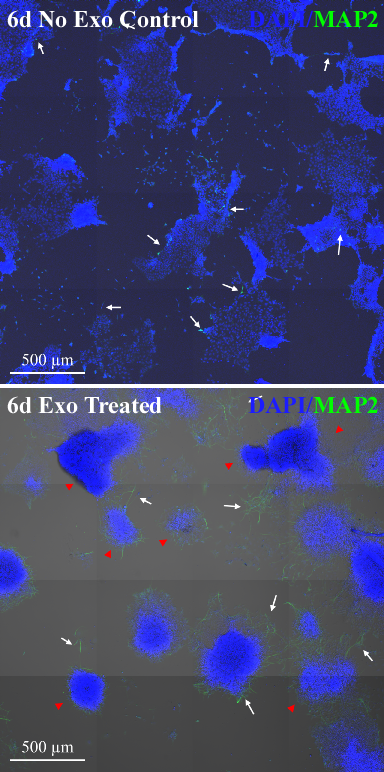


**Supplementary Figure S8c**

Supplement: Supplementary file 14 — Exosome-mediated differentiation of UD-P19 to neurons. Representative tiled images of undifferentiated P19 cells cultured in the absence (Control – No Exo) or presence (Exo Treated) of P19N exosomes for 2 days (a), 4 days (b), and 6 days (c) were captured with a Zeiss confocal microscope using 10X (Plan-Neofluar 10x/0.30) objective. All images were stitched using Stitch feature of Zen software. Cultures terminated at each time point were processed for immunostaining of neurites with MAP2 (green) and nuclei with DAPI (blue). Formation of embryoid bodies following P19N exosome exposure is indicated by red arrowheads and MAP-2 positive cells by white arrows. Scale bar = 500 μm. (DOCX 1564 kb) [file 12015_2023_10512_MOESM8_ESM.docx]

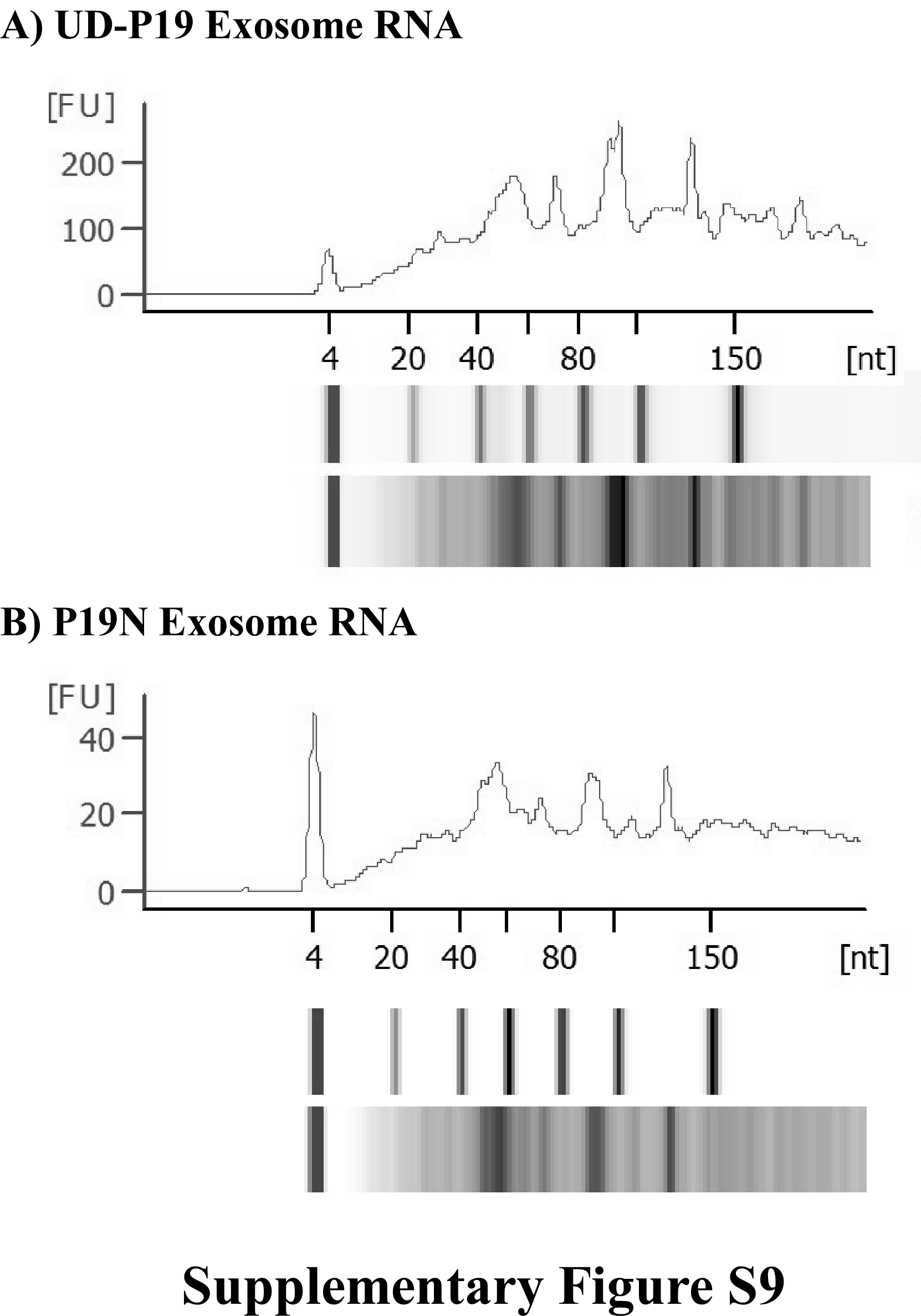

Supplement: Supplementary file 15 — Exosomal RNA analysis on BioAnalyzer. Representative electropherograms were obtained by electrophoresing denatured exosomal RNA in Agilent’s small RNA chip on BioAnalyzer. The 4nt RNA marker was included in every well. Low molecular weight RNA ladder was included in the run. Arbitrary fluorescence units (FU) were plotted as a function of RNA size in nucleotides (nt). a: UD-P19 exosome RNA, b: P19N exosome RNA. (PNG 169 kb) [file 12015_2023_10512_Fig13_ESM.png]

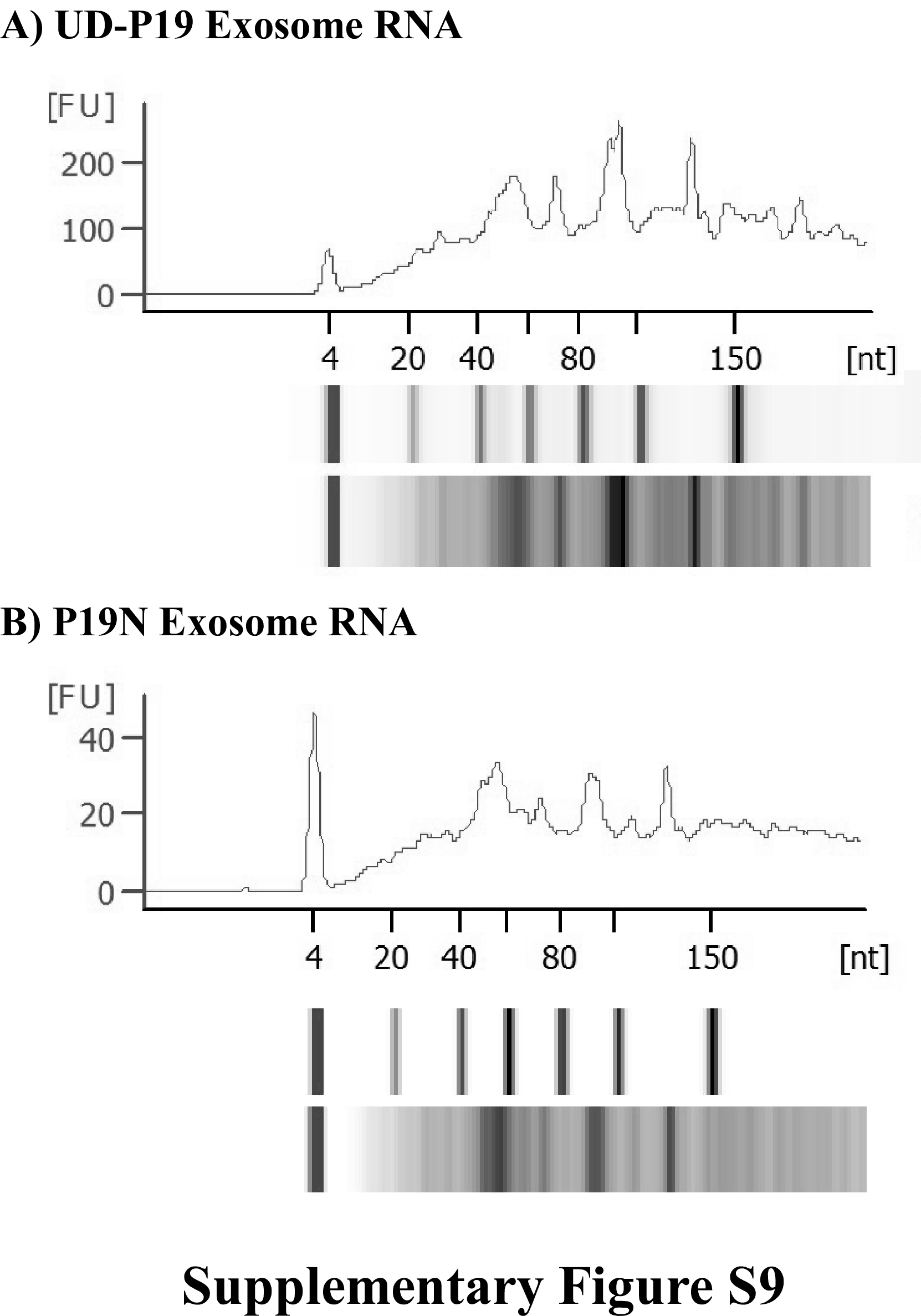

Supplement: Supplementary file 16 — High resolution image (TIF 329 kb) [file 12015_2023_10512_MOESM9_ESM.tif]

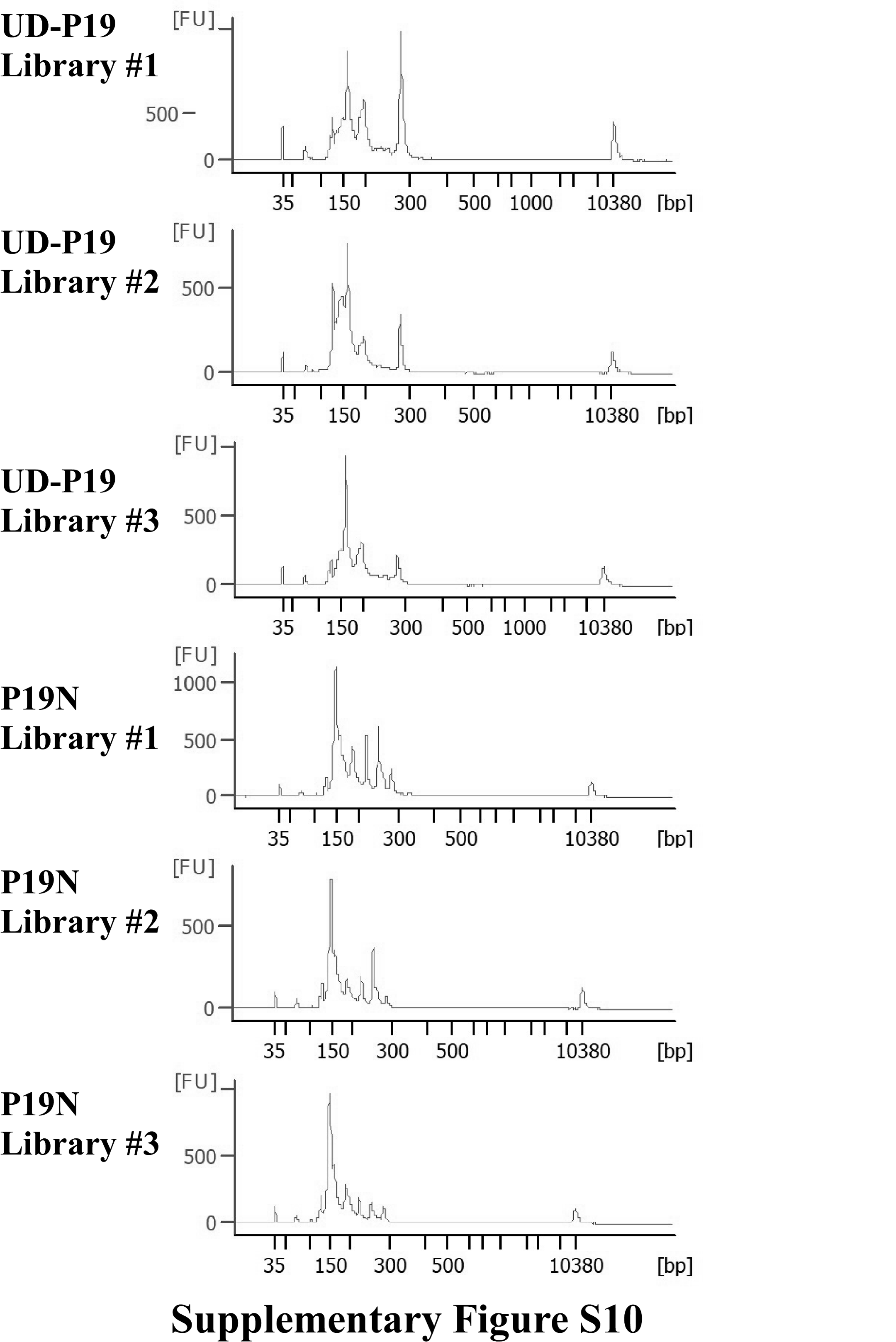

Supplement: Supplementary file 17 — Quality control analysis of barcoded cDNA libraries. Electropherograms of six double-stranded barcoded cDNA libraries (UD-P19 1-3 = three biological replicates of UD-P19 exosomes and P19N 1-3 = three biological replicates of P19N exosomes) obtained on BioAnalyzer using a High Sensitivity DNA chip. Biological replicate for each cell population is indicated on the left. Arbitrary fluorescence units (FU) were plotted as a function of DNA size in base pairs (bp). (PNG 295 kb) [file 12015_2023_10512_Fig14_ESM.png]

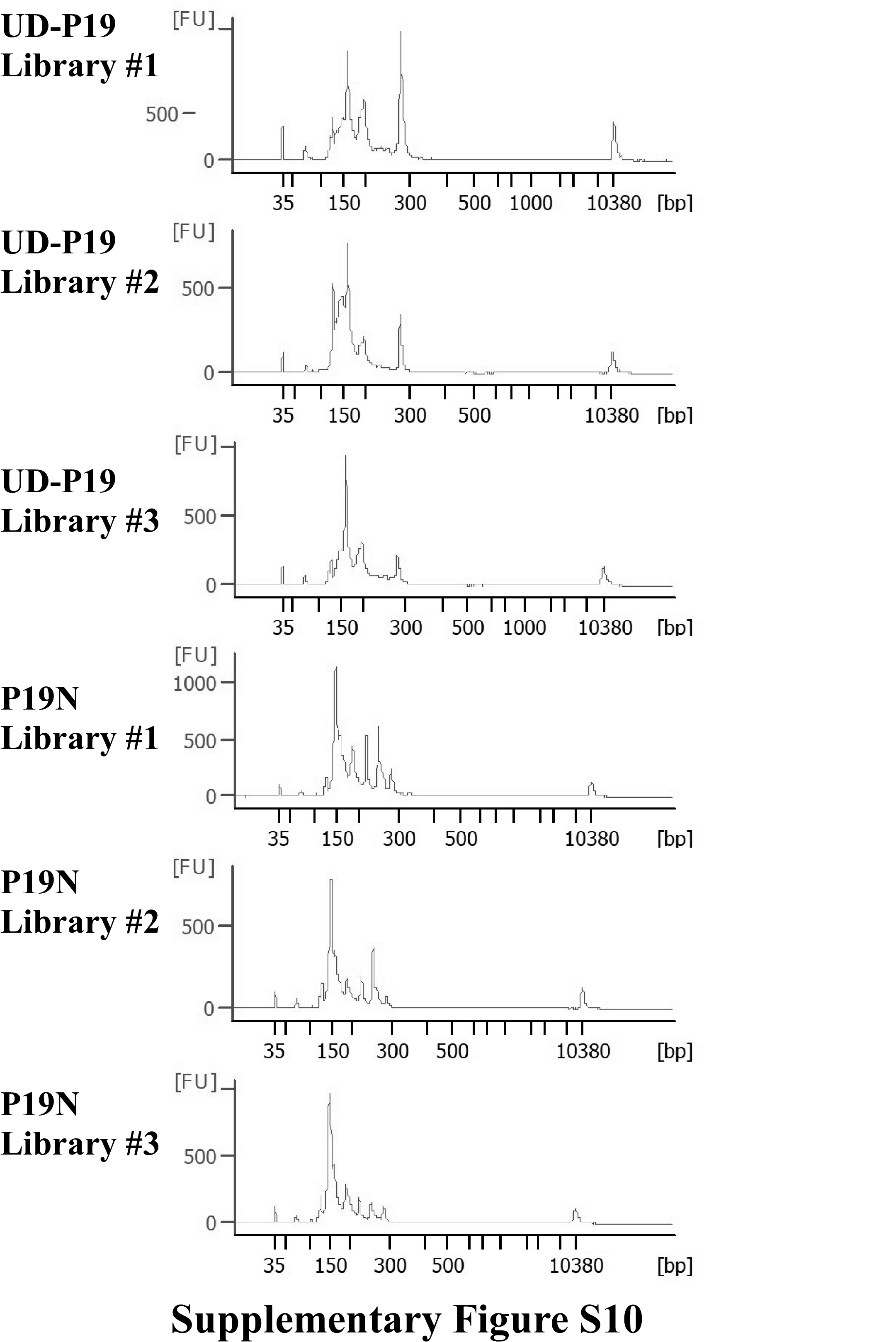

Supplement: Supplementary file 18 — High resolution image (TIF 340 kb) [file 12015_2023_10512_MOESM10_ESM.tif]

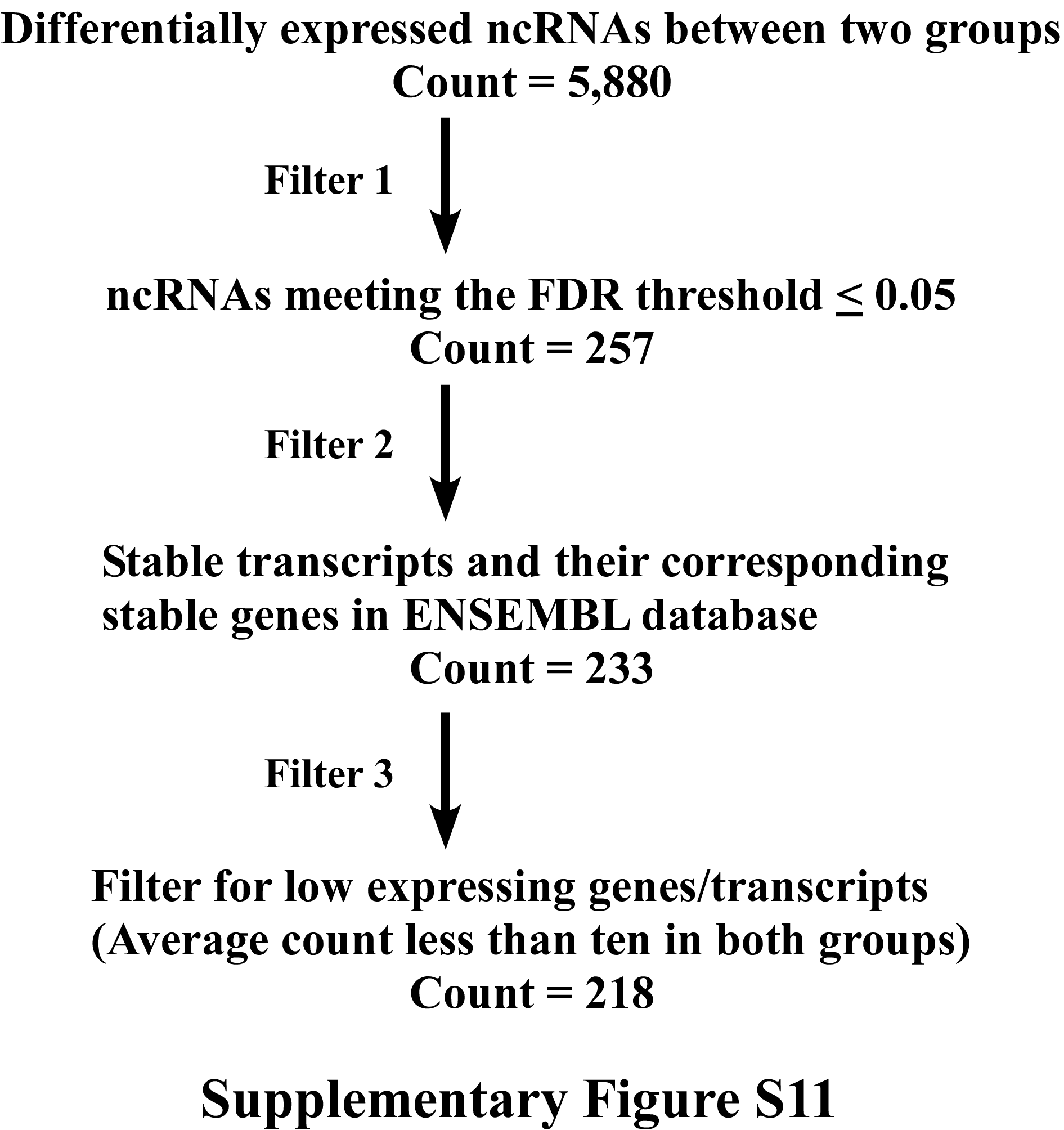

Supplement: Supplementary file 19 — Flow chart showing filtration of differentially enriched/depleted ncRNAs in exosomes. (PNG 69 kb) [file 12015_2023_10512_Fig15_ESM.png]

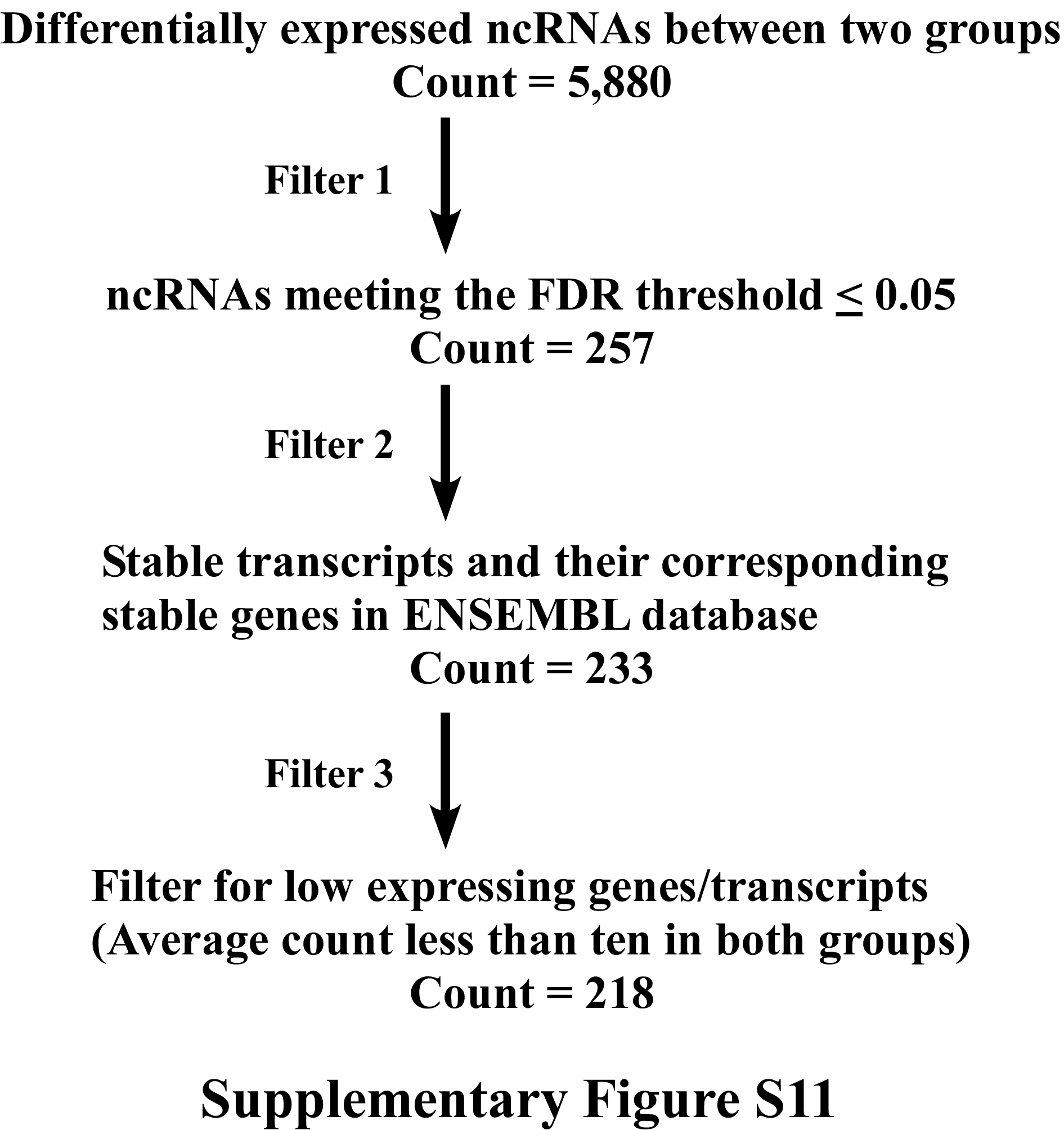

Supplement: Supplementary file 20 — High resolution image (TIF 91 kb) [file 12015_2023_10512_MOESM11_ESM.tif]

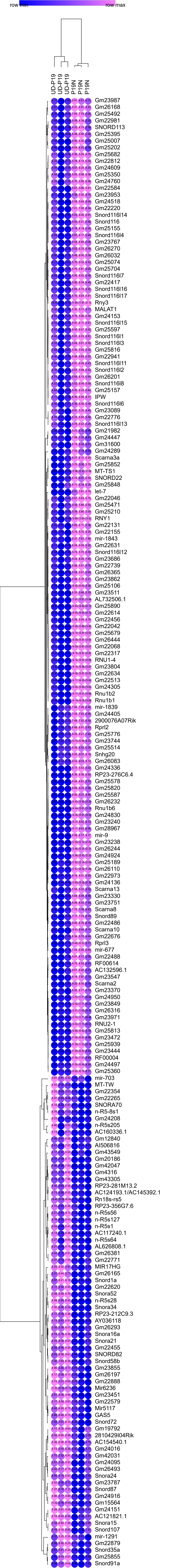

Supplement: Supplementary file 21 — Two-way hierarchical clustering of salient transcripts. The heat map was generated using the log2 transformed expression values of the differentially enriched transcripts purified from UD-P19 and P19N exosomes. To reduce complexity, only 218 transcripts with mean expression values ≥ 10 in UD-P19 or P19N were included in the heat map. The columns represent three biological replicates per parent cell. Each row of circles represents a single transcript. Transcript enrichment is indicated in pink and depletion in blue. The values in the heat map are mapped to colors using the minimum and maximum of each row independently. The numbers in the circles represent the log2 transformed expression values. (PDF 1108 kb) [file 12015_2023_10512_MOESM12_ESM.pdf]
