# Supplementary material for: Exosomes induce neurogenesis of pluripotent P19 cells
Source: Stem Cell Rev Rep. 2023 Feb 22;19(5):1152–76. doi: 10.1007/s12015-023-10512-6 (PMC10366297; doi:10.1007/s12015-023-10512-6)
Supplement: Supplementary file 22 — (DOCX 12 kb) [file 12015_2023_10512_MOESM13_ESM.docx]

**Supplementary Table TS1**: An overview to the size (diameter) of UD-P19 and P19N exosomes determined under hydrated condition by NanoSight (NTA) and under desiccated conditions by transmission electron microscopy (TEM) and atomic force microscopy (AFM).

|  | **Parent Cells for Exosome Isolation** | |
| --- | --- | --- |
| **Technique Employed** | **UD-P19** (average size)  (nm ± Std Dev) | **P19N** (average size)  (nm ± Std Dev) |
| NTA  (mode size) | 143 ± 18 nm | 135 ± 10 nm |
| TEM | 59 ± 16 nm | 42 ± 9 nm |
| AFM | 30 ± 17 nm | 32 ± 15 nm |
