# Supplementary material for: Exosomes induce neurogenesis of pluripotent P19 cells
Source: Stem Cell Rev Rep. 2023 Feb 22;19(5):1152–76. doi: 10.1007/s12015-023-10512-6 (PMC10366297; doi:10.1007/s12015-023-10512-6)
Supplement: Supplementary file 23 — Optimization of sample preparation and Western blot conditions. This table provides details of Western blot conditions optimized and used to detect various proteins in exosomes and cell lysates. Notes to superscripted numbers are provided below the table. (DOCX 14 kb) [file 12015_2023_10512_MOESM14_ESM.docx]

**Supplementary Table TS2**: Optimization of sample preparation and Western blot conditions

| **Antigen/**  **Molecular size** | **UD-P19 Exosome**  **Protein** | **P19N Exosome**  **Protein** | **Cell Lysate^1^**  **Protein Amount** | **Lysis Buffer for solubilizing exosome pellet** | **Resolving gel** | **Stacking gel** | **Exosome lysate storage temp.** |
| --- | --- | --- | --- | --- | --- | --- | --- |
| CD9 (24 kDa) | 30 µg | 30 µg | 30 µg**^2^** | Laemmli buffer | 10% | 4% | -20°C |
| CD63 (25 kDa)**^3^** | 50 µg | 30 µg | 50-100 µg | RIPA buffer**^4^** | 10% | 4% | -70°C |
| CD81 (22-26 kDa) | 30 µg | 30 µg | 30 µg**^4^** | Laemmli buffer | 10% | 4% | -20°C |
| Flotilin-1 (52 kDa) | 40 µg | 30 µg | 30 µg | Laemmli buffer | 8% | 3.5% | -20°C |
| Tsg101 (45 kDa) | 40 µg | 30 µg | 30 µg | Laemmli buffer | 8% | 3.5% | -20°C |
| Cytochrome C (15 kDa) | 40 µg | 30 µg | 30 µg | Laemmli buffer | 8% | 3.5% | -20°C |
| Argonaute-2**^5^** (97 kDa) | N/A | 100 µg | 100 µg | Buffer A**^6^** | 6% | 2.5% | -20°C |
| Dicer1**^5^** (219 kDa) | N/A | 100 µg | 100 µg | Buffer A**^6^** | 6% | 2.5% | -20°C |

Superscript numbers: 1= cell lysate (nuclei-free) from UD-P19, P19N, and fetal cortical neurons (FCN) were included as controls for Western blotting. 2= 20 µg FCN lysate and 30 µg of P19N and UD-P19 lysates were loaded on the same gel as exosome lysates. 3= CD63 is a 25kDa protein but multiple bands were detected on Western blot due to heavily glycosylation of CD63. 4 = Exosomes suspended in RIPA buffer were incubated at 37°C/30 min to dissociate CD63 from exosome membranes. Samples were gently vortexed every 5 min during incubation at 37°C. Tubes were wrapped with parafilm prior to incubation at 37°C to prevent evaporation. At the end of incubation, tubes were centrifuged to bring the sample down. CD63 is highly labile and addition of anti-proteases during solubilization was absolutely essential to prevent its degradation. 5= Levels of argonaute-2 and dicer1 were examined only in P19N exosomes; 6= P19N exosomes were suspended in buffer A [50 mM Tris.HCl buffer pH 7.5 containing 150 mM NaCl, 5 mM EDTA, 0.5 mM DTT, 0.5% NP40, 1 mM NaF, 1mM AEBSF, 1 mM PMSF] and incubated on ice for 20 min with gentle mixing every 5 minutes. Insoluble fraction was removed by centrifugation at 16,000 g, and clear supernatants recovered were loaded on polyacrylamide gel.
